# Label-free protein-structure-sensitive live-cell microscopy for patient-specific assessment of myeloma therapy

**DOI:** 10.1038/s41551-025-01443-3

**Published:** 2025-07-14

**Authors:** Francesca Gasparin, Marlene R. Tietje, Eslam Katab, Aizada Nurdinova, Tao Yuan, Andriy Chmyrov, Nasire Uluç, Dominik Jüstel, Florian Bassermann, Vasilis Ntziachristos, Miguel A. Pleitez

**Affiliations:** 1 Institute of Biological and Medical Imaging, Helmholtz Zentrum München, Neuherberg, Germany; 2 Chair of Biological Imaging at the Central Institute for Translational Cancer Research (TranslaTUM), School of Medicine and Health, Technical University of Munich, Munich, Germany; 3 Department of Medicine III, Klinikum rechts der Isar, Technical University of Munich, Munich, Germany; 4 Center for Translational Cancer Research (TranslaTUM), Technical University of Munich, Munich, Germany; 5 Institute for Computational Biology, Helmholtz Zentrum München, Neuherberg, Germany; 6 Cancer Consortium (DKTK) and German Cancer Research Center (DKFZ), Heidelberg, Germany; 7 Bavarian Centre for Cancer research (BZKF), Munich, Germany; 8 German Cancer Consortium (DKTK) and German Cancer Research Center (DKFZ), Heidelberg, Germany; 9 DZHK (German Centre for Cardiovascular Research), partner site Munich Heart Alliance, Munich, Germany

**Keywords:** Imaging and sensing, Infrared spectroscopy, Myeloma, Biomedical engineering, Protein folding

## Abstract

The efficacy of drug therapy in multiple myeloma is conventionally assessed by whole-cell-population methods, serum analysis of light chains and monoclonal antibodies, immunofixation electrophoresis, or by flow cytometry of bone marrow aspirates and biopsies. These methods provide relevant information on the presence of specific immunoglobulins at high sensitivity and specificity but require a large number of cells, involve long and laborious sample preparation steps, and provide only tumour bulk information. Here we develop a single-cell imaging technique requiring a reduced number of primary cells for longitudinal evaluation of patient-specific treatment and assessment of treatment heterogeneity. By exploiting the mechanistic action of proteasome inhibition and in synergy with the label-free protein-structure specificity of mid-infrared optoacoustic microscopy, we present a technology that facilitates longitudinal evaluation of myeloma treatment and a patient’s heterogeneous response. Detecting optical-generated ultrasound waves that intensify with optical absorption, this technology allows observation of proteins in living cells with high sensitivity. Specifically, we use intermolecular β-sheet formation as a biomarker for cell viability during therapy and apply it to assess drug-treatment performance in multiple myeloma patients.

## Abstract (web summary)

A live-cell microscopy method assesses patient-specific efficacy of myeloma treatment in real time at the single-cell level.

## Main

The cellular ubiquitin-proteasome system (UPS) is responsible for the degradation of dysfunctional intracellular proteins, such as misfolded proteins resulting from α-helix to intermolecular β-sheet conformational transitions^1^. When accumulated, misfolded proteins lead to cytotoxic aggresome formation that eventually triggers apoptosis^1^. In cancer therapy, inhibition of the UPS to induce apoptosis is a common strategy for the treatment of multiple myeloma, amyloid light-chain (AL) amyloidosis, or non-Hodgkin’s lymphoma subtypes, among others^2^. In clinical research, the efficiency of proteasome inhibitors is typically assessed by determination of cell viability. Viability of treated cells, particularly in a clinical research context, is assessed by cell proliferation using fluorescence-activated cell sorting (FACS) assays^3,4^ in combination with detection of the formation of endoplasmic reticulum (ER) stress-inducing agents and pro-apoptotic proteins by western blot (WB)^5,6^. Although these methods allow detection of specific cell-death pathways, they only deliver snapshots of data on whole-cell populations, require a large number of cells (from tens of thousands to millions) per measurement^7,8^, and involve long and laborious multistep sample preparation. In a clinical setting, response to multiple myeloma treatment is routinely assessed by analysis of light chains of monoclonal antibodies in serum, by immunofixation electrophoresis, or by microscopic or flow cytometric analysis of bone marrow aspirates and biopsies^9,10^. These methods provide clinically relevant information on the presence of specific immunoglobulins, but they also require several thousand cells, involve long laboratory procedures that prevent real-time evaluation of therapy response in patients, and only provide tumour bulk information. In contrast, a single-cell imaging technique sensitive to protein structure, requiring only a minimum number of cells and capable of rapidly assessing the heterogeneous response to proteasome inhibitors, would greatly ease treatment evaluation in individual patients using directly extracted myeloma cells. This capability is important because myeloma cells are extracted through painful biopsies, are difficult to isolate in abundance, and are challenging to maintain in culture outside the bone marrow microenvironment^11–13^.

Several imaging techniques have been employed to observe changes in protein conformation and folding states in living cells, such as single-molecule fluorescence resonance energy transfer (sm-FRET)^14^, fluorescence lifetime imaging FRET (FLIM FRET)^15^ and in-cell nuclear magnetic resonance (NMR)^16,17^. Although these techniques afford detection of structural and biochemical features at atomic resolution and are highly specific for tracking protein folding, they require bulky fluorescent or isotopic labels that may interfere with the study of proteins, induce cytotoxicity, and (in the case of fluorophores) are prone to photobleaching^18,19^. In addition, circular dichroism has been applied to detect secondary structure conformational changes in antimicrobial peptides, from random coils to α-helices during interaction with living bacterial cells^20^; however, the analytes in the cell buffer absorb UV light leading to interference with protein signal readouts^21^.

Alternatively, vibrational spectroscopy and imaging has demonstrated label-free sensitivity to protein secondary structures, typically by detecting C = O stretching vibrations of the protein backbone in the amide I band (1,700–1,600 cm^−1^)^22,23^. For instance, stimulated Raman scattering (SRS) microscopy, in combination with deuterium labels or deuterated water, has been used to visualize protein aggregates in cells^24^; however, due to low sensitivity in the amide I region(1–10 mM)^22,25^, no specificity to a particular secondary structure element was demonstrated. In addition, mid-infrared (mid-IR) spectroscopy has been proven to be a valuable tool for analysis of protein secondary structure and is commonly used to study purified protein solutions^26^. However, usage of mid-IR spectroscopy/imaging for protein structure analysis in living cells is limited by the strong optical absorption of water (*µ*_a_: ~742,600 M^−1^ cm^−1^ at 1,650 cm^−1^)^27,28^. Water opacity has been minimized in protein spectral imaging of living cells by using a cellular medium enriched by D_2_O in combination with synchrotron radiation^29^ and thin path lengths of ≤10 µm^30^. However, the signal-to-noise ratio (SNR) remains low due to the negative-contrast detection mechanism of conventional mid-IR spectroscopy/imaging wherein only (unabsorbed) transmitted or trans-reflected photons are detected. This represents a paradox in conventional mid-IR spectroscopy/imaging where higher optical absorption of an analyte of interest results in a lower number of photons available for its detection. Moreover, subjecting cells to high-intensity radiation (as when using synchrotron excitation) and tight spatial confinement can cause photo-damage, substantially alter the cells’ morphology and promote deviation from their native physiological responses^31,32^. Recently, optical photothermal infrared microscopy (OPTIR) was applied to image β-sheet structures and protein aggregation in primary neurons at high spatial resolution and contrast^33–35^. However, due to the low thermo-optic coefficients of water and protein (*dn*/*dT*: ~9 × 10^−5^ and ~1.4 × 10^−4^, respectively), OPTIR applies high-intensity tightly focused probe-beam irradiation for detection, which could induce thermal stress and photo-oxidative damage of cells over long exposure^36,37^.

Unlike conventional optical microscopy, mid-infrared optoacoustic microscopy (MiROM) is a positive-contrast modality that allows label-free spectral imaging of biomolecules in living cells by detecting optically generated ultrasound waves that increase in intensity as optical absorption increases^38^. We have previously demonstrated that MiROM affords higher sensitivity than Raman microscopy in the fingerprint spectral region (1,800–900 cm^−1^)^25,38^ but, until now, proteins have been detected mainly by using the amide II band (1,600–1,500 cm^−1^) mainly associated with C-N stretching and N-H bending vibrations. However, the amide II spectral band does not provide sufficient specificity to protein secondary structure and, thus, cannot be used for efficient detection of intermolecular β-sheet conformational transitions^23^.

We hypothesized that the positive-contrast mechanism of MiROM in combination with partial substitution of D_2_O for H_2_O in the cell medium would yield the necessary sensitivity and specificity in the amide I region for label-free structure-specific detection of protein contrast in living cells without the need for intense radiation and ultra-thin path lengths. Access to structure-specific protein contrast in living cells allows label-free monitoring of proteasome inhibition response in cancer therapy by exploiting the spectral features of intermolecular β-sheet structures as an intrinsic biomarker of cell viability. Thus, aiming for clinical translation, we applied MiROM to monitor intermolecular β-sheet formation upon myeloma therapy with lenalidomide (LEN) (immunomodulatory drug) and bortezomib (BTZ) (proteasome inhibitor) in an immortalized multiple myeloma cell line (MM1.S) as well as in primary myeloma cells aspirated from patients.

Our results demonstrate that MiROM can assess patient-specific efficacy of myeloma treatment in real time at a single-cell level in small cell populations—representing the clinical value of live-cell mid-infrared microscopy in assessing drug response at single-cell level in multiple myeloma (MM). Evaluation of myeloma therapy by direct observation of changes in protein secondary structure instead of via reduction of monoclonal protein concentration in serum could contribute to the acceleration and optimization of the efficacy of medical interventions. Moreover, achieving detection of treatment response at a single-cell level allows assessment of heterogeneity in primary myeloma cells, which could provide crucial therapeutic information (such as individual sensitivity to treatment) for optimizing personalized intervention^39^.

**Fig. 1:**
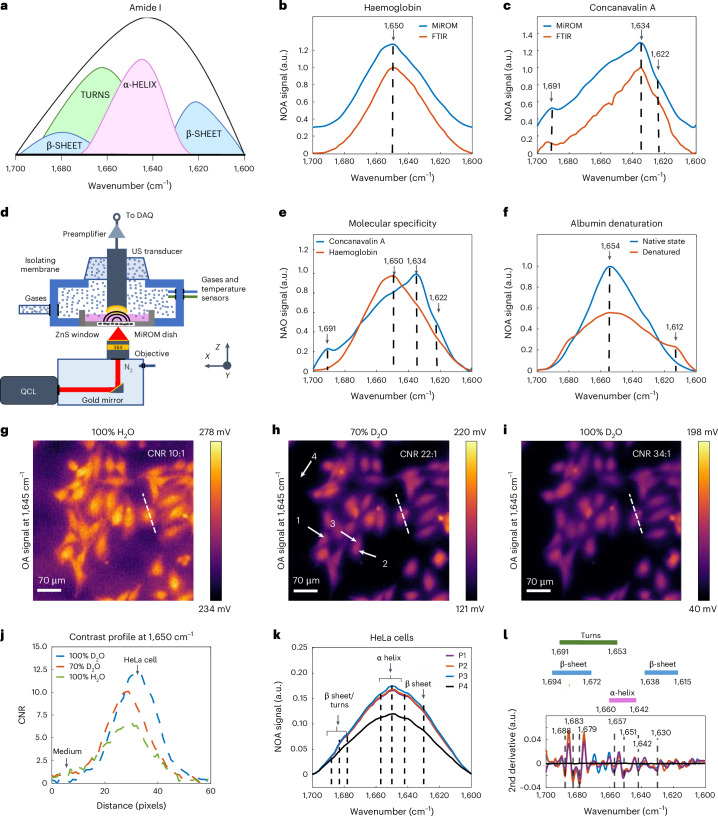
Label-free detection of the secondary structure of proteins. **a**, Illustration of the amide I mid-IR spectrum of proteins (1,700–1,600 cm^−1^) showing the absorption bands of different secondary structures. Modified from ref. ^35^. **b**, Comparison of mid-IR absorption spectra of haemoglobin measured by MiROM (blue line) and standard ATR-FTIR spectroscopy (red line). Haemoglobin is an α-helix protein with a characteristic band at 1,650 cm^−1^ (ref. ^38^). **c**, Comparison of mid-IR absorption spectra of concanavalin A measured by MiROM (blue line) and standard ATR-FTIR spectroscopy (red line). Concanavalin A is a homotetramer protein with a β-sheet structure and characteristic absorption bands at 1,622, 1,634 and 1,691 cm^−1^ (ref. ^28^). **d**, Comparison of mid-IR absorption spectra of haemoglobin (red line) and concanavalin A (blue line) measured by MiROM. **e**, Comparison of mid-IR absorption spectra of albumin in the native state (primarily α-helix, blue line) and denatured state (primarily β-sheet, red line). The vertical dashed lines indicate the α-helix absorption peak at 1,654 cm^−1^ and the intermolecular β-sheet absorption peak at 1,612 cm^−1^. **f**, Schematic diagram of the MiROM imaging system. A tunable quantum cascade laser (QCL) provides excitation for optoacoustic imaging, while a focused ultrasound (US) transducer is used for signal readout. **g**–**i**, MiROM micrographs of live HeLa cells imaged at 1,645 cm^−1^ in cell media composed of different H_2_O/D_2_O proportions. **j**, Contrast-to-noise ratio (CNR) profile of HeLa cells in **g**–**i**. The contrast profile in 100% D_2_O is 3.4 times higher than that in pure H_2_O, while the contrast profile in 70% D_2_O is 2.2 times higher than that in pure H_2_O. The observed apparent shift in the contrast profile is caused by cell movements between measurements. **k**, Normalized spectra in the amide I region of live HeLa cells in 70% D_2_O medium acquired at the points highlighted by the arrows in **h**. The vertical dashed lines indicate the turn, α-helix and β-sheet secondary structure absorption peaks (see Supplementary Table 2). **l**, Each coloured line represents the second derivative of the spectrum in the same colour in **k**. The scheme above the plot details the values corresponding to the different secondary structures according to the literature^43^. *n* = 3 independent experiments. OA, optoacoustic; NOA, normalized optoacoustic.

Spectroscopic analysis of the amide I region (1,700–1,600 cm^−1^) allows differentiation of protein secondary structures because the optical absorption in this spectral region is sensitive to the conformations of peptide bonds (Fig. 1a)^40^. First, we demonstrated MiROM’s ability to distinguish between different secondary structural motifs using protein solutions with different proportions of α-helices and β-sheets (haemoglobin with ~75% α-helices and concanavalin A with ~54% β-sheets)^41^. As common practice in conventional mid-IR spectroscopy of proteins, D_2_O was used as a solvent instead of H_2_O to minimize optical absorption in the amide I region^42^ (see Supplementary Fig. 1 and Methods sections ‘Protein solutions’ and ‘Imaging system description’ for details). As expected, and in good agreement with measurements by attenuated total reflection Fourier-transform-infrared (ATR-FTIR) spectroscopy (see Supplementary Table 1), the spectrum of haemoglobin featured a characteristic absorption band at 1,650 cm^−1^ typical of α-helices (Fig. 1b,d)^43^, while the spectrum of concanavalin A featured a main absorption band at 1,634 cm^−1^ as well as two minor side bands at 1,622 and 1,691 cm^−1^, all of which could be attributed to β-sheet structures (Fig. 1c,d)^28^. Sensitivity to differentiate protein secondary structures was further demonstrated in other proteins, such as Aβ peptide 1–42, insulin and lysozyme (see Supplementary Fig. 2 and ‘Protein solutions’ in Methods). Moreover, we demonstrated MiROM’s ability to detect conformational changes in proteins by recording amide I spectra before and after heat-induced denaturation of albumin (see Fig. 1e and ‘Protein solutions’ in Methods). Albumin is known to undergo irreversible denaturation above 70 °C, and its conformational change induced by temperature has been extensively studied and characterized by mid-IR spectroscopy^44^. In agreement with literature, we observed a peak at 1,654 cm^−1^ representative of α-helices when albumin was in its native state^44^. This peak at 1,654 cm^−1^ flattens upon albumin denaturation, leading to a peak at 1,612 cm^−1^. Importantly, the presence of a spectral feature at 1,612 cm^−1^ is associated with the formation of intermolecular β-sheets and is a specific marker of albumin’s irreversible denaturation^44^.

We next applied MiROM to analyse the amide I region of HeLa cells in D_2_O/H_2_O medium to investigate its sensitivity to protein secondary structure in living cells. To guarantee a controlled environment for the cells’ growth, we outfitted MiROM with a mini-incubator, which maintains optimal humidity and flux of oxygen and CO_2_ (Fig. 1d). HeLa cells were first imaged at 1,645 cm^−1^ in cell media composed of different D_2_O/H_2_O percentages: 0:100% (Fig. 1g), 70:30% (Fig. 1h) and 100:0% (Fig. 1i). Notably, we observed that MiROM could image HeLa cells even in 100% H_2_O, albeit with a lower contrast than in media with higher D_2_O percentages (Fig. 1g–j and Supplementary Fig. 3). Using 100% D_2_O afforded an enhancement in contrast-to-noise ratio (CNR, see Methods for details) up to 3.4 times compared with using pure H_2_O, while using 70% D_2_O afforded an enhancement of 2.2 times (CNR 34:1 in 100% D_2_O, Fig. 1i; CNR 22:1 in 70% D_2_O, Fig. 1h; CNR 10:1 in pure H_2_O, Fig. 1g**;** see Supplementary Fig. 3 and ‘HeLa cells’ in Methods). As shown in Fig. 1g–i, higher contrast was obtained when imaging HeLa cells in 100% D_2_O as compared with cell media with lower D_2_O percentages. We demonstrated cell viabilities up to 93.8% in HeLa cells exposed to 70% D_2_O and up to 85.2% when HeLa cells were exposed to 90% D_2_O over a 24 h exposure time (see Supplementary Fig. 4 and ‘HeLa cells’ in Methods for details). However, long exposure to D_2_O can cause cell toxicity, therefore we reduced exposure time to 2 h maximum in all experiments. MiROM was then used to extract spectra from several intracellular locations in live HeLa cells (white arrows in Fig. 1h). Inspection of the spectral content at these locations revealed several features associated with protein secondary structure in the amide I region, such as α-helices (1,660–1,642 cm^−1^)^23^, β-sheets (1,638–1,615 cm^−1^ and 1,694–1,672 cm^−1^)^23^ and turns (1,691–1,653 cm^−1^)^23^ (see Fig. 1k,l and Supplementary Table 2). As expected and in good agreement with observations of lyophilised cells by FTIR spectroscopy combined with synchrotron excitation^45^, the spectral content obtained with MiROM at different cell locations indicates a homogeneous distribution of protein structures (Δ*λ* = 2 cm^−1^ maximum spectral deviation, Fig. 1l).

**Fig. 2:**
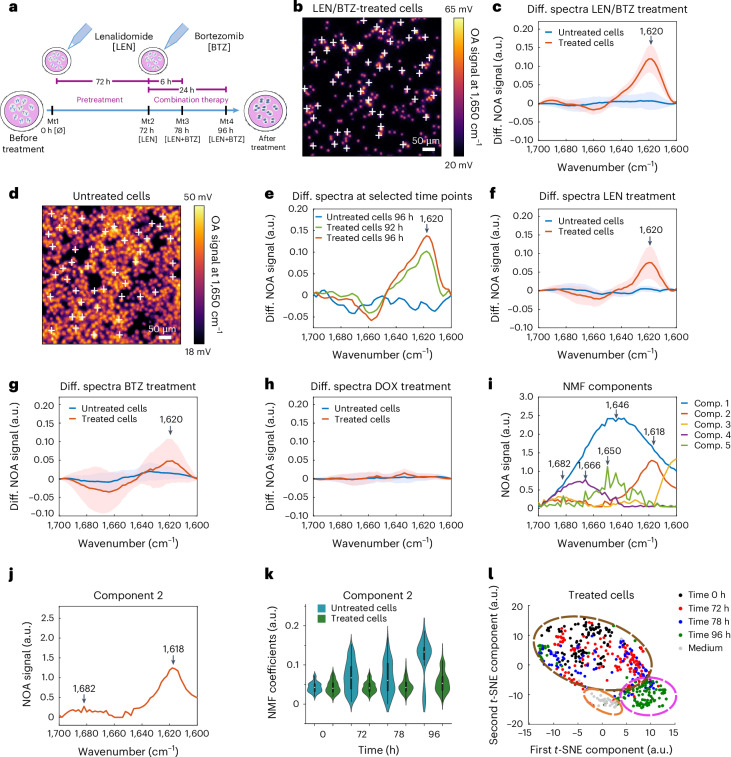
Monitoring protein misfolding in myeloma cells during synergic and individual lenalidomide and bortezomib treatment. **a**, Schematic diagram of LEN and BTZ treatment in myeloma cells. **b**, Amide I map of multiple myeloma cancer cells (MM1.S) pretreated with 10 μM of LEN for 72 h and subsequently treated with 100 nM of BTZ for 24 h, then imaged at 1,650 cm^−1^ (Mt4). The white crosses indicate the myeloma cells selected for spectral analysis. **c**, Differential spectra of the LEN/BTZ-treated myeloma cells shown in **b** (red line) and the untreated myeloma cells shown in **d** (blue line) at 96 h. The spectrum in red shows a band at 1,620 cm^−1^, assigned to an intermolecular β-sheet of misfolded proteins, which was not found in untreated cells. Spectra are presented as mean ± s.d. **d**, Amide I map of untreated myeloma cells imaged at 1,650 cm^−1^ after being in culture for 96 h. The white crosses indicate the myeloma cells selected for spectral analysis. **e**, Differential spectra of LEN/BTZ-treated MM1.S cells at different time points (green, 92 h; red, 96 h). The differential spectrum of untreated cells is shown in blue. MiROM can detect the effect of proteasome inhibition after 92 h of LEN and BTZ combined treatment (*n* = 5 independent experiments). **f**, Differential spectra of MM1.S cells treated with 25 μM of LEN for 144 h compared to untreated cells maintained in culture for 144 h. LEN-treated cells show formation of the intermolecular β-sheet structure, which is not present in the untreated cells (*n* = 3 independent experiments). Spectra are presented as mean ± s.d. **g**, Differential spectra of MM1.S cells treated with 100 nM of BTZ for 18 h compared to untreated cells maintained in culture for 18 h. BTZ-treated cells show formation of the intermolecular β-sheet structure, which is not present in the untreated cells (*n* = 3 independent experiments). Spectra are presented as mean ± s.d. **h**, Differential spectra of MM1.S cells treated with 1 μM of doxorubicin (DOX) for 2 h compared to untreated cells maintained in culture for 2 h. The intermolecular β-sheet structure is not visible in either differential spectra (untreated and DOX-treated cells, *n* = 3 independent experiments). Spectra are presented as mean ± s.d. **i**, NMF components extracted from spectral data (*n* = 700 spectra). **j**, Component 2 represents intermolecular β-sheet secondary structures (1,682–1,618 cm^−1^). **k**, Violin plots from kernel density estimate the time evolution coefficients of NMF component 2 obtained from 700 spectra from 5 independent experiments acquired in LEN/BTZ-treated cells and untreated cells. Component 2 increased by 174% during the treatment. Boxplot minima and maxima inside the violin plots indicate the interquartile range, the white circles indicate the mean values, and the whiskers indicate the standard deviation (coefficient = 1). **l**, A *t*-SNE map representing the distribution of the 5 components identified in LEN/BTZ-treated myeloma cells. Spectra acquired from the cell medium (dashed orange circle) and spectra acquired from treated cells after 96 h of treatment (dashed magenta circle) are clustered together.

We then set out to apply MiROM to detect conformational transitions of proteins in a cancer cell model (namely, multiple myeloma), where accumulation of misfolded proteins marks a central therapeutic mechanism for approved drugs. The myeloma cell line, MM1.S, produces high amounts of dysfunctional immunoglobulins (paraproteins); treatment with the immunomodulatory drug (IMiD) LEN and the 26S proteasome inhibitor BTZ results in accumulation and aggregation of intracellular misfolded proteins, ultimately leading to cell apoptosis (Fig. 2a)^5,46–49^. Misfolded proteins are rich in intermolecular β-sheet structures^4^, which MiROM can detect using the proteins’ characteristic spectral features in the amide I band. Therefore, we propose detection of intermolecular β-sheet structures as a biomarker for treatment response. For demonstration, individual MM1.S cells in 100% D_2_O medium were arbitrarily selected from MiROM micrographs—at 1,650 cm^−1^ and 500 × 500 µm^2^ field-of-view (FOV)—for spectral analysis in the range between 1,700 and 1,600 cm^−1^ (Fig. 2b). The spectral analysis was conducted before treatment, and then at different time points after LEN/BTZ treatment; LEN/BTZ are drugs commonly used in MM therapy (see ‘MM1.S’ in Methods for details)^50,51^. Myeloma cells were maintained in 100% D_2_O medium only during MiROM measurements, for a maximum of 2 h. Spectra, acquired from cells by MiROM after 96 h of LEN/BTZ treatment, exhibited broadening of the amide I band towards frequencies characteristic of β-sheets (red line, Supplementary Fig. 5a). Differential spectra (spectral difference before and after treatment, see Methods) further revealed the formation of a prominent spectral feature at ~1,620 cm^−1^ (Fig. 2c shows a representative example from 5 independent experiments). According to literature, this spectral feature is attributed to antiparallel intermolecular β-sheets^40,52,53^ and suggested the presence of misfolded protein aggregates. A similar spectral band, indicating protein aggregation, was found (in a separate study by other authors) in amyloid fibrils containing β-sheets in phantoms and in human tissues^23,40,54^. As a control, cells neither treated with LEN nor with BTZ were spectrally analysed with MiROM in the same way (Fig. 2d and Supplementary Fig. 5b). No spectral changes were observed in untreated cells over time (Fig. 2c and Supplementary Fig. 5b). Spectral analysis at treatment points earlier than 96 h reveals the appearance of the intermolecular β-sheet band after 84 h of treatment (Supplementary Fig. 6), with the band becoming more intense and pronounced 8 h later (at 92 h, Fig. 2e). No intermolecular β-sheet band was observed at earlier treatment times (at 78 h, Supplementary Fig. 6). Interestingly, when studying the individual effects of LEN^5^ or BTZ^5,55^ treatment, we observed a similar formation of β-sheet intermolecular secondary structures in myeloma cells as a treatment response. This is shown in Fig. 2f,g, where a prominent band at 1,620 cm^−1^ appeared after MM1.S cells were subjected to 25 μM LEN (treatment response expressed in 50% of cells after 96 h and in 100% of cells after 144 h) or to 100 nM BTZ (treatment response expressed in 80% of cells after 18 h; see Supplementary Figs. 7 and 8, and ‘MM1.S’ in Methods). MiROM’s detection of intermolecular β-sheet formation at 1,620 cm^−1^ upon LEN treatment alone suggests that (similarly to BTZ) LEN might also result in misfolded protein accumulation, supporting combinatory LEN/BTZ treatment as a strategy to increase efficacy^5^. In addition, the reliability of MiROM to detect misfolded protein formation was confirmed by observation of the intermolecular β-sheet at 1,620 cm^−1^ in HeLa cells treated with the proteasome inhibitor MG132 for 22 h (Supplementary Fig. 9). Similarly to BTZ, MG132 causes accumulation of immature proteins in the ER, activating intrinsic pathways that ultimately lead to apoptosis^56,57^.

The specificity of MiROM for detecting the mechanistic action of LEN/BTZ treatment response was further demonstrated by conducting experiments on MM1.s cells treated with doxorubicin (DOX). DOX is a chemotherapeutic agent that induces apoptosis by damaging DNA^58^ without causing a direct formation of misfolded protein aggregates. Since proteins are not affected directly by DOX, no intermolecular β-sheet band is expected to form after treatment of myeloma cells with DOX. This was demonstrated in the differential spectra analysis of DOX-treated MM1.s cells (Fig. 2h, Supplementary Fig. 10 and ‘MM1.S’ in Methods). The comparison between the differential spectra of cells with or without DOX treatment revealed the absence of the β-sheet intermolecular band, providing evidence of MiROM’s specificity to the mechanism of action for LEN and BTZ (Fig. 2h).

To obtain a more detailed picture of spectral changes during MM1.S treatment (than with the differential spectra analysis; Fig. 2c), the acquired (LEN/BTZ treated and untreated) single-cell spectra (~700 spectra from 5 independent experiments) were analysed by regularized non-negative matrix factorization (NMF). The number of spectral components necessary to suitably represent the dataset was estimated via principal component analysis (PCA) (see Methods section ‘MM1.S’ and Supplementary Fig. 11a). Figure 2i shows the 5 main components obtained by NMF analysis of spectra from untreated and LEN/BTZ-treated cells. Component 1, centred at 1,646 cm^−1^, describes a common feature within the spectra acquired, associated with the cell medium. Component 2, at 1,618 cm^−1^, is associated with the presence of intermolecular β-sheets (see Fig. 1a). Component 3 describes the emission profile of the laser (Supplementary Fig. 11b). Component 4, at 1,666 cm^−1^, and component 5, at 1,650 cm^−1^, can be associated with the presence of α-helix structures (see Fig. 1a) and are influenced by the exchange between the hydrogen in the atmosphere and the deuterium in the medium (see Supplementary Fig. 11c). In Fig. 2j, component 2 shows a second band at 1,682 cm^−1^ (characteristic of intermolecular β-sheet structures^40^), which was not clearly visible from the differential spectra analysis. According to literature, the band at 1,682 cm^−1^ is weaker than that at 1,620 cm^−1^ (ref. ^40^), and therefore more difficult to detect by MiROM alone without the application of NMF analysis. In Fig. 2k, violin plots show the time evolution of component 2 for LEN/BTZ-treated and untreated cells. In treated cells, component 2 increases by 174% after 96 h of treatment with LEN/BTZ (violin plots in blue, Fig. 2k), confirming the increase of intermolecular β-sheet secondary structures as observed by the differential spectral analysis (Fig. 2c). The time evolution of component 2 describes the trend of both intermolecular β-sheet bands (1,682 and 1,620 cm^−1^). We noticed (Supplementary Fig. 11f,g) that the absorption bands of components 4 and 5 are overlapping with the absorption band at 1,682 cm^−1^, therefore, we consider that the decreasing dynamics of component 4 (~33%) and 5 (~25%) suppresses the intensity increase at 1,682 cm^−1^. This might explain why we could not detect the band at 1,682 cm^−1^ when a simple difference spectral analysis is applied (Fig. 2c). Moreover, the decreasing dynamics of components 4 and 5 generates a dip at ~1,650 cm^−1^ in the differential spectra, suggesting a reduction in α-helix structures. As expected, in untreated cells, the time evolution of component 2 does not show substantial changes (violin plots in green, Fig. 2k), confirming the absence of intermolecular β-sheet formation. This result is in agreement with our differential spectral analysis (Fig. 2c) that shows no change in the spectra of untreated cells. A stable trend for component 5 in untreated cells suggests retention of α-helix structures in the cells’ proteins; for components 1–3, the time evolution changes are negligible (see Supplementary Fig. 11d–g for more details). For better visualization, PCA and NMF analysis of the measured spectra yielded a two-dimensional *t*-distributed stochastic neighbour embedding (2D *t*-SNE) map illustrating the contributions of the 5 molecular spectral components to each spectrum (see Methods section ‘MM1.S’). The *t*-SNE map of LEN/BTZ-treated cells in Fig. 2l shows three separate groupings: (1) spectra acquired from 0 to 78 h (dashed brown circle), (2) spectra acquired after 96 h (dashed magenta circle) and (3) spectra of the medium (dashed orange circle). The grouping of points on the *t*-SNE maps reflects similarities in protein secondary structure. This means that the spectra taken after 96 h of combinatory treatment with LEN/BTZ were spectrally different from those obtained at any earlier time points. In a similar fashion, the *t*-SNE map of untreated cells (Supplementary Fig. 11h) shows only 2 groups: one composed of cell spectra acquired at all time points (magenta circle) and a second group composed of spectra of the media (orange circle); these groupings indicate that there are no changes in secondary structure and are in agreement with the differential spectral analysis (Fig. 2c).

The presence of the spectral band at 1,620 cm^−1^, obtained by differential spectral analysis (Fig. 2c) as well as by NMF analysis (Fig. 2i–k), clearly indicates formation of intermolecular β-sheets in proteins of MM1.S cells when treated with LEN or BTZ individually or in combination; this demonstrates MiROM’s ability to identify intrinsic hallmarks of misfolded proteins and aggresome formation, and treatment response without the use of labels or a large number of cells. Our observations with MiROM are in agreement with observations (by other authors) using immunoblots, apoptosis and 3-(4,5-dimethylthiazol-2-yl)-2,5-diphenyltetrazolium bromide (MTT) cell growth assays, where apoptotic cell death of MM1.S cells occurred in response to LEN or BTZ treatment^5,46–50,59^.

**Fig. 3:**
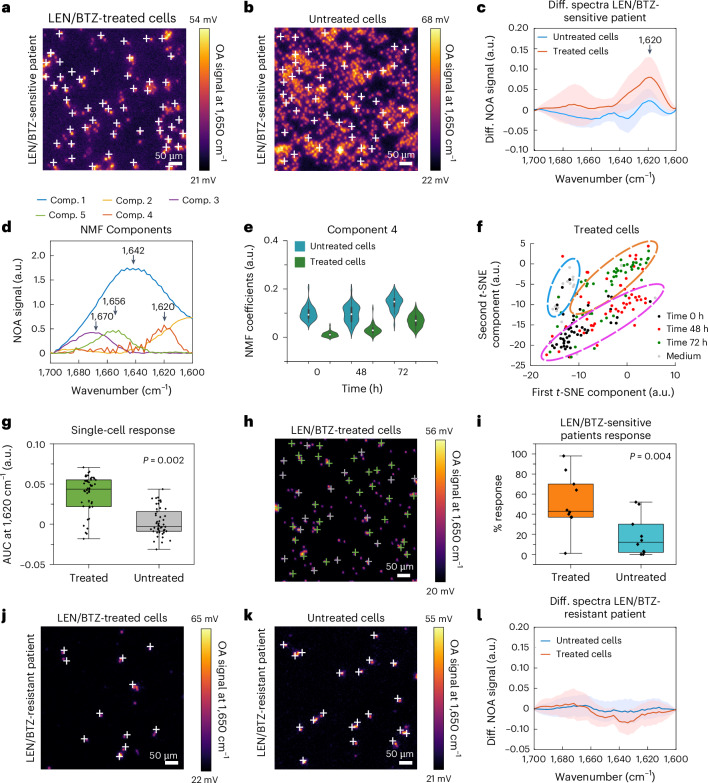
Monitoring protein misfolding of myeloma cells sensitive or resistant to lenalidomide and bortezomib in patients. **a**,**b**, Myeloma cells extracted from the bone marrow of a LEN and BTZ-sensitive patient were plated in two separate dishes (see Methods for details). One dish (**a**) was imaged at 1,650 cm^−1^ after pretreatment for 48 h with 10 μM of LEN and then treatment for 24 h with 100 nM of BTZ. The second dish (**b**) was used as a control (untreated) and imaged at 1,650 cm^−1^ after being in culture for 72 h. **c**, Comparison between the differential spectra of LEN/BTZ-treated (in red) and untreated (in blue) myeloma cells after 72 h. LEN/BTZ-treated cells show the absorption band of an intermolecular β-sheet structure at 1,620 cm^−1^ (*n* = 9 patients). Spectra are presented as mean ± s.d. **d**, NMF components extracted from spectral data in **c** (*n* = 300 spectra). **e**, Violin plots from kernel density estimate the time evolution coefficients of NMF component 4 obtained from 300 spectra acquired in LEN/BTZ-treated cells and untreated cells of one representative patient. Component 4 increased by 44% during the treatment. Boxplot minima and maxima inside the violin plots indicate the interquartile range, the white circles indicate the mean values, and the whiskers indicate the standard deviation (coefficient = 1). **f**, A *t*-SNE map representing the distribution of the 5 components identified in LEN/BTZ-treated myeloma cells. **g**, Boxplot representing the area under the curve (AUC) for the band at 1,638–1,615 cm^−1^ of LEN/BTZ-treated and untreated cells extracted from 100 spectra obtained in one LEN and BTZ-sensitive patient. Boxplot minima and maxima indicate the interquartile range, the centre lines indicate the median values, and the whiskers indicate the standard deviation (coefficient = 1). Outliers are shown as individual points. *P* values from two-sided paired-sample *t*-test. **h**, A total of 32 single cells identified by the AUC analysis show the intermolecular β-sheet band (green cross), while 18 cells do not show the β-sheet band (grey cross). **i**, Boxplots representing the percentage response (%) of LEN/BTZ-treated and untreated cells analysed from 9 independent patients sensitive to LEN and BTZ. Boxplot minima and maxima indicate the interquartile range, the centre lines indicate the median values, and the whiskers indicate the standard deviation (coefficient = 1). Outliers are shown as individual points. *P* values from a two-sided paired *t*-test. **j**,**k**, LEN/BTZ-treated (**j**) and untreated (**k**) myeloma cells extracted from the bone marrow of a LEN and BTZ-resistant patient (r/r patient #1). *P* values from paired-sample *t*-test. **l**, Comparison of differential spectra between LEN/BTZ-treated and untreated myeloma cells in **j** and **k**. Both LEN/BTZ-treated and untreated cells show no spectral band in the region of the intermolecular β-sheet structure (1,638–1,615 cm^−1^), denoting the absence of misfolded protein formation under proteasome inhibition (*n* = 2 patients, r/r patient #1 and 2). Spectra are presented as mean ± s.d.

After demonstrating sensitivity to misfolded protein formation in immortalized cell lines during LEN or BTZ treatment individually or in combination, as a first step towards clinical translation, we applied MiROM to analyse protein misfolding in CD138^+^ purified primary myeloma cells from newly diagnosed myeloma patients (9 patients measured independently). Following the same procedure applied to MM1.S cells, the patients’ cells were measured in 100% D_2_O medium for ~2 h. Figure 3a–f exemplifies results from one representative LEN/BTZ-sensitive myeloma patient (with data from the 9 patients summarized in Supplementary Table 3). Individual cells were arbitrarily selected from MiROM micrographs (at 1,650 cm^−1^, 500 × 500 μm^2^ FOV, Fig. 3a) for spectral analysis (in the amide I region) at different time points: namely, before adding any drug (time 0 h), 48 h after LEN administration (before adding BTZ), and 72 h after LEN and BTZ combinatory treatment (see Methods section ‘MM1.S’). As a control, untreated myeloma cells biopsied from the same patient were measured in the same way (Fig. 3b). Consistent with our results in treated MM1.S cells (Fig. 2c and Supplementary Fig. 5a), spectral analysis of treated patient cells revealed spectral broadening towards lower frequencies (Supplementary Fig. 12a) and the presence of the band at 1,620 cm^−1^, which is characteristic of intermolecular β-sheet structures of misfolded proteins (Fig. 3c). As expected, the spectra of untreated cells did not show a prominent band of intermolecular β-sheet structure (Fig. 3c and Supplementary Fig. 12b), supporting specificity of 1,620 cm^−1^ as an intrinsic spectral marker of treatment response. Moreover, PCA and NMF analysis of patient cells’ spectra identified 5 main components similar to those obtained in the analysis of the MM1.S cell line (Fig. 2i), although in a different order of appearance (see Fig. 3d and Supplementary Fig. 13 for details). In particular, in LEN/BTZ-treated patient cells, component 4 at 1,620 cm^−1^ (indicative of intermolecular β-sheet structures) showed an intensity time evolution that increased (after 72 h of combined treatment) by 44% (Fig. 3e), component 5 at 1,656 cm^−1^ (α-helix indicator) showed decreased intensity by 52% (Supplementary Fig. 13e), while the change over time in the remaining components (1–3) was found to be negligible (Supplementary Fig. 13c–e). Importantly, untreated cells showed negligible change over time for all the 5 components, suggesting unchanged protein secondary structures and supporting our previous observations from differential spectra analysis of untreated MM1.S cells (Supplementary Fig. 13c–f). In addition, the 2D *t*-SNE maps of the 5 molecular spectral components’ coefficients resulted in separated clusters at the different time points after LEN/BTZ treatment (Fig. 3f). Furthermore, there was a unique cluster for the spectral components of untreated cells (Supplementary Fig. 13b), indicating that the spectral components of treated cells changed during treatment while remaining stable in untreated cells.

To take advantage of MiROM’s specificity to intermolecular β-sheet formation as well as its ability for single-cell analysis, we studied the heterogeneity of therapy efficacy in biopsied cells for assessment of treatment response in individual patients. For this purpose, we defined a metric to determine single-cell response on the basis of the spectral content (that is, area under the curve, AUC) in the range specific for intermolecular β-sheet structures (1,638–1,615 cm^−1^) (see Methods section ‘Patient cells’ and Supplementary Fig. 14a–e for details). Cells with AUC larger than the threshold value (defined by the AUC of untreated cells at 0 h) have spectral features indicating development of intermolecular β-sheets and these cells were classified as responsive to treatment. Cells with AUCs smaller than or equal to the threshold value did not show the relevant band and were thus classified as unresponsive. The percentage of responding cells, including details on the number of unresponsive or responsive cells, are summarized in Supplementary Table 3. As exemplified by the boxplots in Fig. 3g (from one representative patient), LEN/BTZ-treated cells have higher AUC values compared with untreated cells (boxplots for all patients shown in Supplementary Fig. 15). Figure 3h shows the distribution of the cells defined as responsive or unresponsive for one representative patient: 32 responsive cells (green marks) and 18 unresponsive cells (grey marks) yielding a positive treatment response in 64% of the total cells. The mean positive treatment response calculated in all the patients analysed was 53% overall. The mean response in untreated cells showed a notably lower value at 20%.

Finally, we applied MiROM to assess therapy response in relapsed/refractory (r/r) patients previously exposed to only LEN or both LEN and BTZ treatment (Fig. 3j–l, from 3 patients as summarized in Supplementary Table 3). Patients with relapsed/refractory myeloma refers to individuals who either did not respond to the treatment at all or experienced a more aggressive recurrence of cancer after a period of remission. In r/r MM patient #1, no or negligible formation of intermolecular β-sheet structures was expected due to its clinically observed refractivity to both LEN and BTZ. Indeed, differential spectra reveals absence of the band indicating intermolecular β-sheet structures at 1,620 cm^−1^ and a single-cell response of 0%, which is comparable to the single-cell response of control untreated myeloma cells of the same patients (see Fig. 3l, and Supplementary Table 3 and Fig. 16a). Second, LEN refractory patient #2 was clinically known to be resistant to LEN but naïve to treatment with BTZ. MiROM’s analysis of MM cells from refractory patient #2 showed absence of intermolecular β-sheet structure, thus suggesting resistance also to BTZ (see Supplementary Table 3 and Fig. 16b). Upon treatment with a BTZ-containing regimen, r/r patient #2 exhibited progression of disease, confirming resistance to BTZ as already predicted by MiROM. Lastly, although diagnosed as LEN refractory, MM cells from r/r patient #3 showed considerable formation of intermolecular β-sheet (in 37% of cells) upon treatment with LEN and BTZ (see Supplementary Table 3 and Fig. 16c), suggesting sensitivity to BTZ. This observation aligned with the clinical outcome of a newly diagnosed setting where the patient was treated with a BTZ-containing regimen and achieved a complete remission from myeloma. Although in the course of the disease, the patient acquired resistance to LEN, MiROM’s measurements were able to observe residual intermolecular β-sheet formation upon treatment, supporting the clinically observed positive sensitivity of the patient to BTZ. Thus, MiROM demonstrated its ability to evaluate not only the positive response of LEN/BTZ-sensitive patients, but also the negative response of LEN/BTZ-resistant patients, predicting (in the case of patients #2 and 3) their resistance or sensitivity to the drugs. Consequently, our results on unresponsive patients provide additional support that MiROM is able to specifically assess LEN and BTZ therapy efficacy in clinical samples via detection of intermolecular β-sheet structures, and that it has potential in terms of response assessment.

Conventional optical microscopy techniques (in particular, those operating in the visible spectral region) lack the sensitivity and specificity for label-free detection of protein secondary structures in living cells. Here we demonstrated that MiROM achieves label-free protein-structure-specific sensitivity in living cells. Instead of applying exogenous markers, MiROM uses the intrinsic spectroscopic signatures of protein secondary structure in the amide I band (1,700–1,600 cm^−1^) as endogenous source of contrast. This ability enabled label-free detection of intermolecular β-sheet formation in primary myeloma cells and MM1.S cells treated with LEN or BTZ individually or in combination. Intermolecular β-sheet formation is a hallmark of misfolded protein accumulation and aggresome formation, which we used as an indicator of cell apoptosis.

Unlike conventional clinical techniques for evaluating therapy response such as serum light chain and protein electrophoresis, immunofixation or flow cytometry of bone marrow biopsies, MiROM evaluations can be performed in real time directly after purification of myeloma cells from the bone marrow. The assessment of myeloma therapy response by observation of intermolecular β-sheet formation instead of detection of reduced monoclonal protein quantities in serum or bone marrow aspirates (with a latency period of 3–4 weeks) could accelerate prediction of myeloma therapy efficacy and outcome. Moreover, MiROM assessment, unlike WB and FACS assays currently used in clinical research to study cell viability^7,8^, requires only a minimum amount of cells and not tens of thousands to millions of cells per measurement point (for example, FACS requires between 30,000 and 50,000 cells and WB requires 1 million cells)^7,8,60^. In addition, MiROM can longitudinally detect drug-induced apoptosis in real time, unlike FACS and WB, which only provide a snapshot of bulk information. Typically, in MM patients, the amount of myeloma cells obtained after purification of a 10 ml liquid biopsy varies between 10,000 to 2 million cells. Therefore, the number of cells collected from each biopsy is frequently insufficient for determination of cell viability after therapy when using WB and FACS assays; this is especially challenging in longitudinal studies where several measurement points are needed to assess drug performance over time. Although additional bone marrow aspirations can be performed to increase cell count from one patient, bone marrow aspiration is painful and associated with stinging and sharp pain, causing discomfort to patients. To increase cell yield, improved purification methods such as immune magnetic bead depletion have been applied to obtain up to 95% of the myeloma cells^12^. Nonetheless, the number of purified cells could still be insufficient for performing longitudinal studies of cell viability. In addition, primary myeloma cell culture and expansion is challenging or even impossible; therefore, this strategy to generate sufficient cultured cells for FACS or WB is not a feasible alternative^13^. Thus, going beyond conventional methods, MiROM offers an alternative to evaluate therapy efficacy in patients’ myeloma cells, allowing us to monitor, presumably for the first time, treatment response to LEN/BTZ in primary patient samples without the use of external labels. The ability to detect apoptotic effects in a small number of cells opens up the possibility of longitudinal assessment of multiple drugs in parallel using cells from the same biopsy, allowing prediction of therapy response for personalized myeloma treatment. In clinical research, evaluation of treatment in a small amount of patient cells using MiROM, as opposed to conventional methods that require a large number of primary myeloma cells^13^, may reduce tedious and painful procedures for the patient. Indeed, MiROM has been demonstrated to be capable of discerning between LEN/BTZ-sensitive and LEN/BTZ-resistant patients by detecting the formation or absence of misfolded protein structures in the amide I region after myeloma treatment. Moreover, achieving detection of therapy response to a single-cell level could allow MiROM to improve understanding of intratumoural heterogeneity by identification of cell subpopulations responsible for causing treatment failure due to drug resistance^39^.

Furthermore, we introduce MiROM as a potential tool for predicting patient responses to drug therapy in MM. By comparing the therapeutic responses of first-diagnosis patients predicted by MiROM with the clinical information reported in Supplementary Table 3, we observed that patients who achieved a good partial response to LEN, BTZ or a combination of both in their induction regimen also showed a positive response (~40%) according to MiROM’s measurement of their bone marrow samples. Moreover, MiROM was able to predict resistance to BTZ therapy for refractory patient #2 and sensitivity to BTZ for refractory patient #3. Refractory patient #2 was known to be clinically resistant to LEN but had unknown sensitivity to BTZ. MiROM measurement of myeloma cells treated with both LEN and BTZ revealed the absence of intermolecular β-sheet bands, thus suggesting resistance to LEN (as expected) and to BTZ (a previously unknown finding). During the course of the treatment, r/r patient #2 was found to be non-responsive to BTZ as already predicted by MiROM. In addition, r/r patient #3 was clinically known to be resistant to LEN but showed formation of intermolecular β-sheet band upon LEN and BTZ treatment when measured by MiROM, thus suggesting sensitivity to BTZ. In the course of the disease, r/r patient #3 was treated with a BTZ-containing regimen, achieving complete remission from myeloma, hence confirming BTZ sensitivity as predicted by MiROM. Therefore, although a dedicated and larger study is needed to validate the ability of MiROM to predict treatment responses for choosing an appropriate therapeutic strategy, the proof of concept in clinical samples presented here showcases the potential of MiROM as an aid to indicate the effect of a certain therapy regimen in real time.

MiROM is a positive-contrast imaging modality that attains sensitivities in the amide I region superior to conventional mid-IR microscopy and spectroscopy methods, which are predominantly negative contrast^38^. This unique characteristic allows MiROM to overcome the limitations encountered by conventional mid-IR spectroscopy and imaging, which can only achieve the detection of protein secondary structures in living cells with the use of high-power synchrotron radiation and ultra-narrow path lengths that perturb cells’ physiology^30–32^. The current sensitivity of MiROM allows detection of signal changes as low as 1% in the amide I region. In this study, we observed a maximum signal change up to 15% in single cells after 72 h of LEN and 24 h of BTZ treatment (that is, after 96 h of combined action) with an average change of ~8%. Further enhancing MiROM’s sensitivity—for example, by using narrower laser pulse durations to increase optoacoustic (OA) efficiency (for example, 5 ns instead of 20 ns as used here)—would allow detecting signal changes smaller than 1%, thus resulting in earlier detection of therapeutic effects. In addition, increasing imaging and spectral acquisition speed will allow analysis in larger cell populations (beyond the 500 × 500 µm^2^ FOV studied here), thus taking full advantage of patient cell extractions and increasing the accuracy of treatment assessment.

Finally, the ability to detect intrinsic protein misfolding and aggregation during MM treatment could also become key in studying therapy efficiency in other diseases treated with proteasome inhibitors, for example, mantle cell lymphoma and AL amyloidosis. In addition, MiROM could also be relevant for studying protein dynamics in other protein misfolding diseases such as Alzheimer’s or Parkinson’s disease, as well as in enabling efficient drug screening in related preclinical studies. Thus, MiROM could contribute to personalized medicine by supporting identification of the most efficient therapy, sparing time and minimizing errors in optimizing patient-specific treatment. Our vision is that MiROM will help physicians to choose the best treatment strategy after simultaneously testing several drug combinations on a single extraction of a patient’s primary cells.

## Methods

### Protein solutions

To investigate the ability of MiROM to distinguish between proteins with different secondary structures, two representative proteins (haemoglobin and concanavalin A) were analysed. The samples were prepared and measured as follows: a 10 mg ml^−1^ D_2_O solution of haemoglobin was prepared by dissolving 500 mg of haemoglobin powder (HiMedia Laboratories) in 50 ml of D_2_O; a 10 mg ml^−1^ D_2_O solution of concanavalin A was prepared by dissolving 200 mg of concanavalin A (Sigma-Aldrich) in 20 ml of D_2_O phosphate buffer. To investigate the ability of MiROM to monitor conformational changes in the secondary structure of proteins, we observed the effect of heat denaturation on a D_2_O solution of albumin. A solution of 50 g l^−1^ D_2_O albumin was prepared by dissolving 125 mg of albumin (Carl Roth) in 25 ml of D_2_O water. The solution was imaged spectrally at different temperatures starting from room temperature (25 °C) where albumin has a native α-helix structure, until above 80 °C where its structure is completely denatured into an intermolecular β-sheet conformation. The heating denaturation process was performed using a stage heating system and the temperature was monitored with a thermometer. All protein solutions were filtered and measured on a custom-made mid-IR dish with a ZnSe window using carbon tape (SPI Suppliers) as a spectral reference for correcting the systematic variation due to factors such as the mid-IR output spectrum, optical components absorption spectrum and so on. Optoacoustic spectra were measured with a resolution of 2 cm^−1^ and an averaging time of 10,000 ms per wavelength between 1,700 and 1,600 cm^−1^. For comparison and validation, a drop of each solution (5 µl) was measured on an ATR-FTIR spectrometer (ALPHA II from Bruker, equipped with a diamond ATR crystal). The FTIR measurements were recorded between 4,000 and 400 cm^−1^. Each spectrum was obtained by averaging 150 scans recorded at a resolution of 2 cm^−1^. The spectra were calibrated by measuring a blank sample. The effect of the buffer solution (D_2_O) on the absorption spectra was removed by dividing the sample’s spectra by an independently measured buffer spectrum. The subsequent spectra were then processed using a Savitzky–Golay smooth filter (polynomial order: 5, frame length, smoothing point: 19) and baseline correction, and were finally normalized to 0–1. The results are reported in Fig. 1b–e and Supplementary Table 1. D_2_O solutions of Aβ peptide 1–42, insulin and lysozyme in Supplementary Fig. 2 were prepared as follows: 1 mg of Aβ peptide 1–42 (BioLegend) was placed in the desiccator for 1 h, dissolved in 0.4 mM of dimethylsulfoxide and diluted to 143 μg ml^−1^ with D_2_O phosphate saline buffer; 1.25 mM solution of insulin (Sigma-Aldrich) was prepared by dissolving insulin powder in a D_2_O solution of NaCl with 7% of HCl (pH 4); 5 mg ml^−1^ of lysozyme was obtained by dissolving the lysozyme powder (BioLegend) in 0.1 M of a D_2_O solution of KH_2_PO_4_. Data analysis was conducted using Matlab 2019b.

### Imaging system description

OA signals are generated using a broadly tunable pulsed quantum cascade laser (QCL, MIRcat, Daylight Solutions). The QCL used had a spectral range of 3.4–11.0 μm and a spectral linewidth of ≤1 cm^−1^ (full width at half maximum, FWHM). The pulse duration of the QCL was set to 20 ns at a repetition rate of 100 kHz, and a reflective objective (×36) with a numerical aperture of 0.5 NA (Newport Corporation) was used to generate a diffraction-limited focal irradiation spot of ~6 µm diameter. OA spectrum was generated in this study by excitation in the amide I range (from 5.88 to 6.25 μm) with a spectral resolution of 2 cm^−1^. Mid-IR OA micrographs were obtained by scanning the sample along the focal plane with a motorized stage (Physik Instrumente) while detecting the OA signal generated at each position and at a selected wavelength with a 25 MHz central frequency focused ultrasound transducer (Sonaxis). A data acquisition system with a sampling rate of 200 mega samples per second (MS s^−1^) was used for OA signal recovery. The transducer and objective were coaxially aligned to share the same focal plane. Interferences from atmospheric CO_2_ and water vapour in the mid-IR spectrum were eliminated by purging a constant and continuous flow of N_2_ along the optical beam path. The average laser power calculated at 1,650 cm^−1^ was 0.53 mW. The time resolution for an FOV of 500 μm × 500 μm (62,500 pixels) using a step size of 2 μm is 11 min per frame, with a moving-stage speed of 2.5 mm s^−1^. The maximum velocity of the stage is 20 mm s^−1^. The spatial (lateral) resolution of the system was experimentally determined to be 5.3 μm at 2,850 cm^−1^, while the theoretical diffraction-limited resolution in the spectral region used in our study is 6 µm. The maximum size of the FOV was determined by the travel range of the motorized stages used, which was a maximum of 52 mm^2^. Data were collected using Matlab 2018b.

### HeLa cells

#### Cell culture

A human cervical adenocarcinoma (HeLa) cell line (ATCC, CCL-2) was used to define protein secondary structure composition in living cells. Cells were cultured in Dulbecco’s modified Eagle medium (DMEM, Life Technologies) supplemented with 10% fetal bovine serum (FBS, Merck), 1% l-glutamine and 1% penicillin–streptomycin (Life Technologies). Cells were grown in a humidified incubator at 37 °C and 5% CO_2_, and were passed through trypsin digestion every 4 days to avoid reaching full confluence.

#### Spectral imaging analysis

HeLa cells were plated in a custom-made mid-IR dish and cultured until 80% confluence. To interrogate the effect of D_2_O on the absorption spectra and on the image contrast of living HeLa cells in the amide I range, three DMEM media with different percentages of D_2_O were prepared by dissolving DMEM powder (Sigma-Aldrich) in MilliQ water (0% D_2_O), in 70% D_2_O (Sigma-Aldrich) and 30% MilliQ water, and finally in 100% D_2_O. All solutions were supplemented with 3.7 g l^−1^ sodium bicarbonate (Sigma-Aldrich), 10% FBS, 1% l-glutamine and 1% penicillin–streptomycin. Culture medium was exchanged before measurements. HeLa cell micrographs (FOV of 500 μm × 500 μm) were imaged at 1,645 cm^−1^, while HeLa cell spectra were acquired between 1,700 and 1,600 cm^−1^ (step-size resolution of 2 cm^−1^ and an averaging time of 10,000 ms per wavelength) in all three media. Absorption spectra were smoothed and normalized with baseline correction (Fig. 1k), and corresponding second derivative spectra were obtained by interpolation and the Savitsky–Golay method (Fig. 1l). Data analysis was conducted using Matlab 2019b.

#### Contrast-to-noise ratio

CNR is defined as the intensity difference between a point in the sample (OA_S_) and a point in the background/cell medium (OA_ref_) divided by the peak-to-peak amplitude of the noise level.

1 $${\rm{CNR}}=\left|{\rm{{OA}}_{S}}-{\rm{{OA}}_{{ref}}}\right|/{\rm{{Noise}}_{{PkPk}}}$$

The CNR was calculated in all three HeLa cell micrographs (Fig. 1g–i). The noise level was 4.4 mV (pure water), 4.5 mV (70% D_2_O) and 4.7 (100% D_2_O). Data analysis was conducted using Matlab 2019b.

#### Contrast profiles of HeLa cells

The plot profiles of HeLa cells in all three media were obtained with ImageJ and normalized as follows:

2 $${\rm{{NOA}}_{S}}=\left|{\rm{{OA}}_{S}}-{\rm{{OA}}_{{ref}}}\right|/{\rm{{OA}}_{{ref}}}$$

The normalized optoacoustic signal of the cells (NOA_**S**_) was obtained as the intensity difference between the sample optoacoustic signal (OA_S_) and the point in the background/cell medium with the lowest contrast (OA_ref_) divided by the same point in the background (Fig. 1j). Data analysis was conducted using Matlab 2019b.

#### Cell viability

To interrogate the toxic effect of D_2_O on cell viability, HeLa cells cultured in media composed of different percentages of D_2_O were analysed with flow cytometry after staining with propidium iodide (PI/RNase, BD Biosciences), which easily penetrate dead or damaged cells (see Supplementary Fig. 3). Cell viability was determined in three different culture media created by dissolving DMEM powder in 50, 70 and 90% D_2_O and 50, 30 and 10% MilliQ water, respectively. DMEM medium created using 100% MilliQ water was used as a control. The media were supplemented with 3.7 g l^−1^ sodium bicarbonate, 10% FBS, 1% l-glutamine and 1% penicillin–streptomycin as previously described. Cell viability was measured at different time points: 0, 1, 3, 6, 9 and 24 h after exchanging the control (100% MilliQ) medium with a medium partially composed of D_2_O. Cells were then washed with phosphate-buffered saline (PBS) and incubated with PI/RNase (10 μg ml^−1^) for 20 min in the dark. Cell suspensions were analysed using a Cytoflex LX (Beckman Coulter) flow cytometer and analysed using FlowJo software.

### MM1.S

#### Cell culture

Human myeloma cells MM1.S (ATCC, CRL-2974) were cultured in RPMI-1640 cell media (Gibco) supplemented with 10% FBS and 1% penicillin–streptomycin. Cells were grown in a humidified incubator at 37 °C and 5% CO_2_, and were split 1:5 every 4 days to avoid cells reaching full confluence.

#### Spectral imaging analysis

MM1.S cells were plated in polylysine (Sigma-Aldrich) coated custom-made mid-IR dishes with a 70–80% confluence. Before measurement, the RPMI medium was exchanged with a D_2_O medium obtained by dissolving RPMI-1640 powder (Gibco) in 100% D_2_O. The medium was supplemented with 2 g l^−1^ sodium bicarbonate (Sigma-Aldrich) and 20% FBS. To study the spectral changes caused by misfolded protein formation and consequent apoptosis, we treated myeloma cells for 72 h with 10 µM LEN (Sigma-Aldrich) and with 100 nM BTZ (Velcade, Ratiopharm) (Fig. 2b). For comparison, myeloma cells not treated with any drug were also plated in the mid-IR dish before the measurement (Fig. 2d). LEN solution (10 μM) was prepared by dissolving 250 mg of LEN powder in dimethylsulfoxide (Sigma-Aldrich), while 2.5 mg ml^−1^ of BTZ (Velcade) was diluted in sterile water to a final concentration of 100 nM. After medium exchange, MM1.S cells were imaged at 1,650 cm^−1^ at different time points: before addition of any drug (Mt1), after 72 h of treatment with LEN (Mt2), after 78 h of LEN treatment and 6 h of treatment with BTZ (Mt3), and after 96 h of treatment with LEN and 24 h of treatment with BTZ (Mt4). At each time point, the amide I spectra of the cells were acquired (1,700–1,600 cm^−1^, step size 2 cm^−1^, averaging time 10,000 ms per wavelength). All the spectra acquired were normalized with baseline correction (Supplementary Fig. 4). In the differential spectral analysis (Fig. 2c), the normalized average spectrum acquired at time 0 (Mt1) was subtracted from the normalized average spectrum acquired at 96 h (Mt4) (and at the other time points) to detect the appearance of absorption bands assigned to misfolded proteins. For individual drug studies, MM1.S were treated with 25 μM LEN for 144 h and with 10 μM BTZ for 18 h. Data analysis was conducted using Matlab 2019b.

#### Doxorubicin treatment

To confirm the ability of MiROM to selectively detect intermolecular β-sheet structure as a hallmark of misfolded protein formation and consequent apoptosis, MM1.S cells were treated with DOX, a chemotherapy drug that causes an arrest of the cell cycle through DNA intercalation. MM1.S cells were treated with 1 μM DOX for 2 h in 100% D_2_O and spectrally imaged as previously described. MM1.S untreated cells were also spectrally imaged for 2 h in 100% D_2_O for comparison (Supplementary Fig. 10).

#### Computational analysis

To illustrate MiROM’s sensitivity to protein structure composition, MM1.S spectra were processed with several data analysis tools using the scikit-learn Python package (v.3.7.1)^61^.

#### Principal component analysis

To estimate the dimensionality of the MiROM spectral dataset, PCA was performed. More than 99.97% of the data variance was explained by the first 5 principal components.

#### Non-negative matrix factorization

Following the dimensionality estimate of the PCA, the spectral data were linearly decomposed into 5 components via NMF^62^. The method was chosen to obtain physically reasonable features that could be interpreted as partial absorption spectra due to their non-negativity. The NMF was regularized with a combination of Frobenius norm and sparsity promoting L^1^-norm terms. More precisely, the following optimization problem was solved to achieve a decomposition *X* = *WH*, where the rows of the matrix *X* contain the acquired MiROM spectra, the rows of the matrix *H* contain the 5 NMF components, and the rows of the matrix *W* contain the NMF coefficients of the spectra in *X*:

3 $$\begin{array}{l}(W,H):={\rm{arg }}\mathop{\min }\limits_{(W{\prime} ,H{\prime} )}\frac{1}{2}{\Vert X-W{\prime} H{\prime} \Vert }_{F}^{2}+\alpha \rho {\Vert W{\prime} \Vert }_{1}\\\,+\,\alpha \rho {\Vert H{\prime} \Vert }_{1}+\frac{1}{2}\alpha (1-\rho ){\Vert W{\prime} \Vert }_{F}^{2}+\frac{1}{2}\alpha (1-\rho ){\Vert H{\prime} \Vert }_{F}^{2}.\end{array}$$

The NMF model was initialized with the non-negative double singular value decomposition method^63^. A coordinate descent solver was used for the optimization. The tolerance rate and the maximum number of iterations were set to 10^−5^ and 2,000, respectively. The regularization parameters *α* = 10^−2^ and *ρ* = 10^−3^ were chosen using the L-curve^64^.

#### *t*-distributed stochastic neighbour embedding

To obtain an interpretable visualization of the 5D NMF coefficient data, we performed a nonlinear dimensionality reduction with *t*-SNE to embed the data in 2D space^65^.

### Patient cells

Multiple myeloma cells were biopsied from the bone marrow of 12 patients sensitive or resistant to lenalidomide and bortezomib treatment. Myeloma cells were biopsied according to the guidelines indicated in the ethics vote 672/21-S issued by the TUM (Technical University Munich) University Hospital ethics committee on 17 November 2021. Informed consent was obtained from all patients before measurements. Freshly extracted myeloma cells were plated in polylysine-coated mid-IR dishes in RPMI-1640 medium supplemented with 20% FBS, as previously described. To investigate the effect of LEN/BTZ treatment on protein secondary structure in patients’ cells, myeloma cells were imaged at 1,650 cm^−1^ (FOV 500 μm × 500 μm, 2 μm step size, 50 average) after treatment with 10 μM LEN for 72 h and with 100 nM BTZ for 24 h. At each time point, spectra in the amide I region of 50 cells were acquired (1,700–1,600 cm^−1^, step size 2 cm^−1^, averaging time 10,000 ms per wavelength). All spectra acquired were normalized with baseline correction (Supplementary Fig. 12), and the differential spectra (Fig. 3d) were calculated by subtracting the normalized average spectra acquired at time 0 from the normalized spectra acquired at 72 h. Data analysis was conducted using Matlab 2019b.

#### Least-squares method

To calculate the treatment response of single cells, we fitted spectra obtained from patients’ cells with the cell medium spectrum by using the least-squares method. Differential spectra were calculated by subtracting the mean value of the fitted spectrum acquired at 0 h from the spectra at 72 h. We determined the AUC of the differential spectra in the spectral region between 1,638 and 1,615 cm^−1^ and compared it with the AUC of untreated cells, using as a threshold the AUC mean value of untreated cells measured at 0 h. Data are available in the repository (10.5281/zenodo.15755913) (ref. ^66^). Data analysis was conducted using Matlab 2019b.

### Reporting summary

Further information on research design is available in the Nature Portfolio Reporting Summary linked to this article.

## Supplementary information

Supplementary Information Supplementary Figs. 1–16 and Tables 1–3.

Reporting Summary

## Figures and Tables

**Extended Data Fig. 1:**
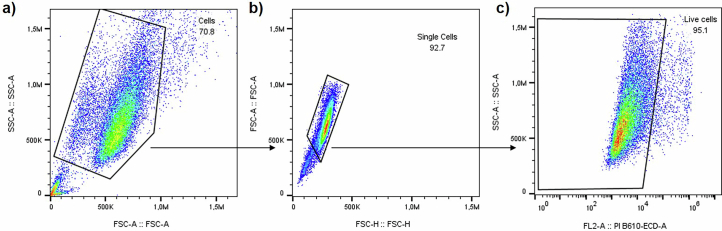
Fluorescence activated cell-sorting (FACS) sequential gating strategy for determining cell viability upon exposure to deuterated water (D_2_O) cell medium. **(a)** SSC-A vs FSC-A (P1) for HeLa cells exposed to 90% of D_2_O. (**b**) FSC-A VS FSC-H (P2) of singlets. (**c**) SSC-A vs PI B610-ECD-A.

## Data Availability

The data that support the findings of this study are available from the corresponding authors upon reasonable request.
