# Supplementary material for: Label-free protein-structure-sensitive live-cell microscopy for patient-specific assessment of myeloma therapy
Source: Nat Biomed Eng. 2025 Jul 14;10(1):56–68. doi: 10.1038/s41551-025-01443-3 (PMC12823395; doi:10.1038/s41551-025-01443-3)
Supplement: Supplementary file 1 — Supplementary Figs. 1–16 and Tables 1–3. [file 41551_2025_1443_MOESM1_ESM.pdf]

nature biomedical engineering

Supplementary information

https://doi.org/10.1038/s41551-025-01443-3

Label-free protein-structure-sensitive live-cell microscopy for patient-specific assessment of myeloma therapy

In the format provided by the authors and unedited

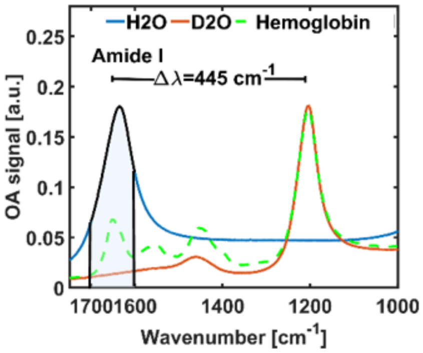

Supplementary Figure 1. Effect of D_2O in the amide I band. Attenuated Total Reflection Fourier Transform InfraRed (ATR-FTIR) spectra of H_2O (blue line), D_2O (red line) and a D_2O albumin solution (green line). The absorption band of H_2O overlaps with the amide I band of the hemoglobin solution, preventing its detection. The absorption band of D_2O is shifted at 1205 cm^{-1}. The use of D_2O clarifies amide I absorption region and allows protein detection. The amide I absorption region is highlighted in light blue. OA – Optoacoustic.

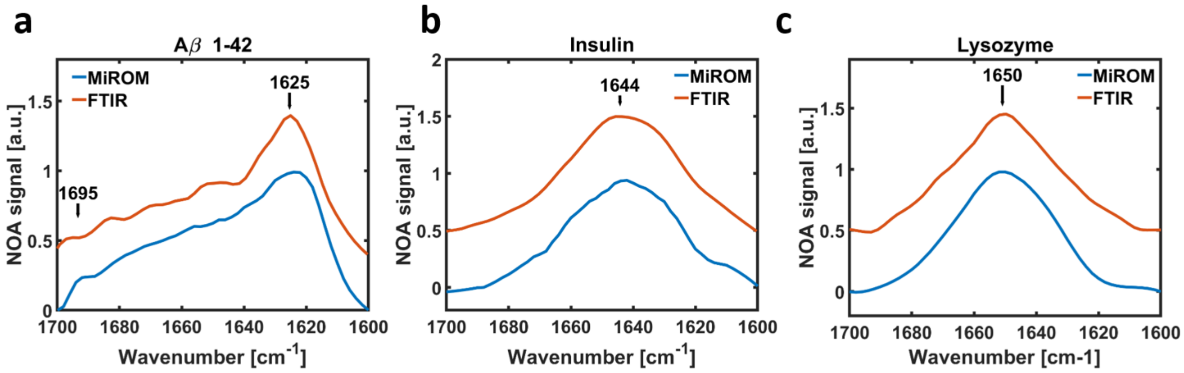

Supplementary Figure 2: **a)** Comparison of mid-IR absorption spectra of Aβ1-42 peptide (dissolved in deuterated water) measured by MiROM (in blue) and standard ATR-FTIR spectroscopy (red line). Aβ1-42 peptides aggregate into oligomers with antiparallel β-sheet structures with characteristic bands at 1625 cm^{-1} and 1695 cm^{-1}. **b)** Comparison of mid-IR absorption spectrum of bovine pancreas insulin (dissolved in deuterated water) measured by MiROM (in blue) and standard ATR-FTIR spectroscopy (in red). Insulin shows a band at 1644 cm^{-1}, which represents intermediate dimers while transitioning from α-helixes to β-sheets. **c)** Comparison of mid-IR absorption spectrum of lysozyme (dissolved in deuterated buffer solution) measured by MiROM (in blue) and standard ATR-FTIR spectroscopy (in red). Insulin shows a band at 1650 cm^{-1}, which represents α-helixes structures. MiROM: mid-infrared optoacoustic microscopy, NOA: Normalized Optoacoustic, ATR-FTIR: Attenuated Total Reflectance Fourier-transform infrared.

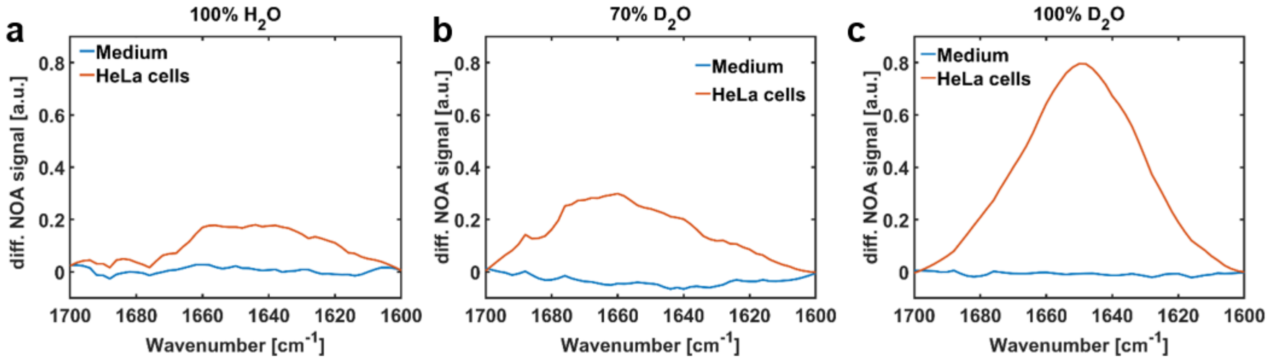

Supplementary Figure 3. Amide I absorption spectra in HeLa cells. a) Amide I absorption spectrum of HeLa cells imaged in a medium composed of 100% H_2O. The absorption peak of the cells is two times higher than the baseline (medium). **b)** Amide I absorption spectrum of HeLa cells imaged in 70% D_2O. The absorption peak of the cells is three times higher than the baseline (medium). **c)** Amide I absorption spectrum of HeLa cells imaged in 100% D_2O. The absorption peak of the cells is eight times higher than the baseline (medium). (n=3 independent experiments). NOA – Normalized Optoacoustic.

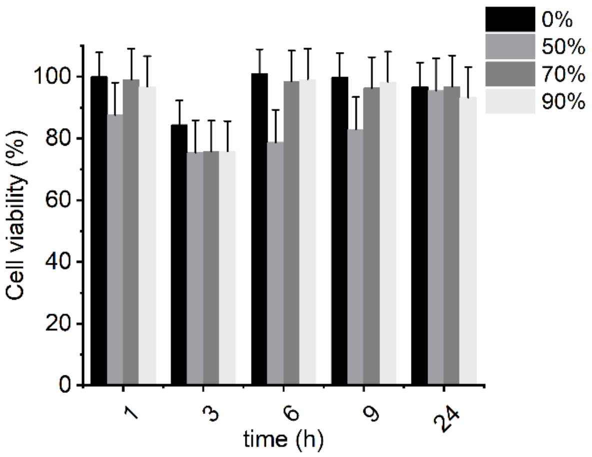

Supplementary Figure 4. Cell viability. a) Propidium iodide (PI) staining for cell viability assessment using Fluorescence-Activated Cell Sorting (FACS) analysis of HeLa cells. HeLa cells were cultured in media containing different percentages of D_2O (0, 50, 70 or 90%) and measured at different time points. The use of deuterated water does not appear to change cell viability substantially compared to controls.

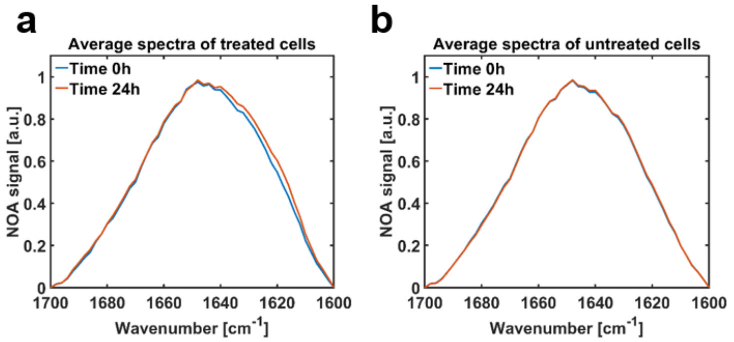

Supplementary Figure 5. Raw spectra from myeloma cells (MM1.S). a) Comparison between the normalized average spectra of 50 myeloma cells at time point 0 (before lenalidomide (LEN) and bortezomib (BTZ) administration) and after 96 hours of LEN/BTZ treatment. **b)** Comparison between the normalized average spectra of 50 untreated myeloma cells at time point 0 and after 96 hours in culture. In (a), the spectrum acquired at 96 hours shows an enlargement towards lower frequencies compared to the spectrum at time 0, while in (b) there is no difference between the average spectra acquired at time 0 and 96 hours after the medium exchange. NOA – Normalized Optoacoustic.

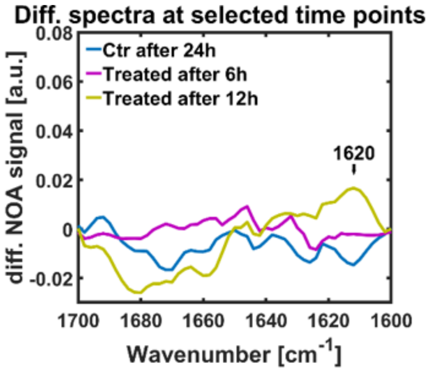

Supplementary Figure 6. Differential spectra of myeloma cells treated with lenalidomide (LEN) and bortezomib (BTZ). Comparison between the differential spectra of LEN/BTZ treated (in violet and green) and untreated (in blue) myeloma cells acquired in the amide I region at different time points (78h and 84h). The band at 1620 cm^{-1}, corresponding to intermolecular β-sheet structures which accumulate in misfolding proteins during the proteasome inhibition treatment, is barely detectable after 84h of treatment. All cells were imaged using a coupling medium composed of 100% deuterated water. NOA – Normalized Optoacoustic.

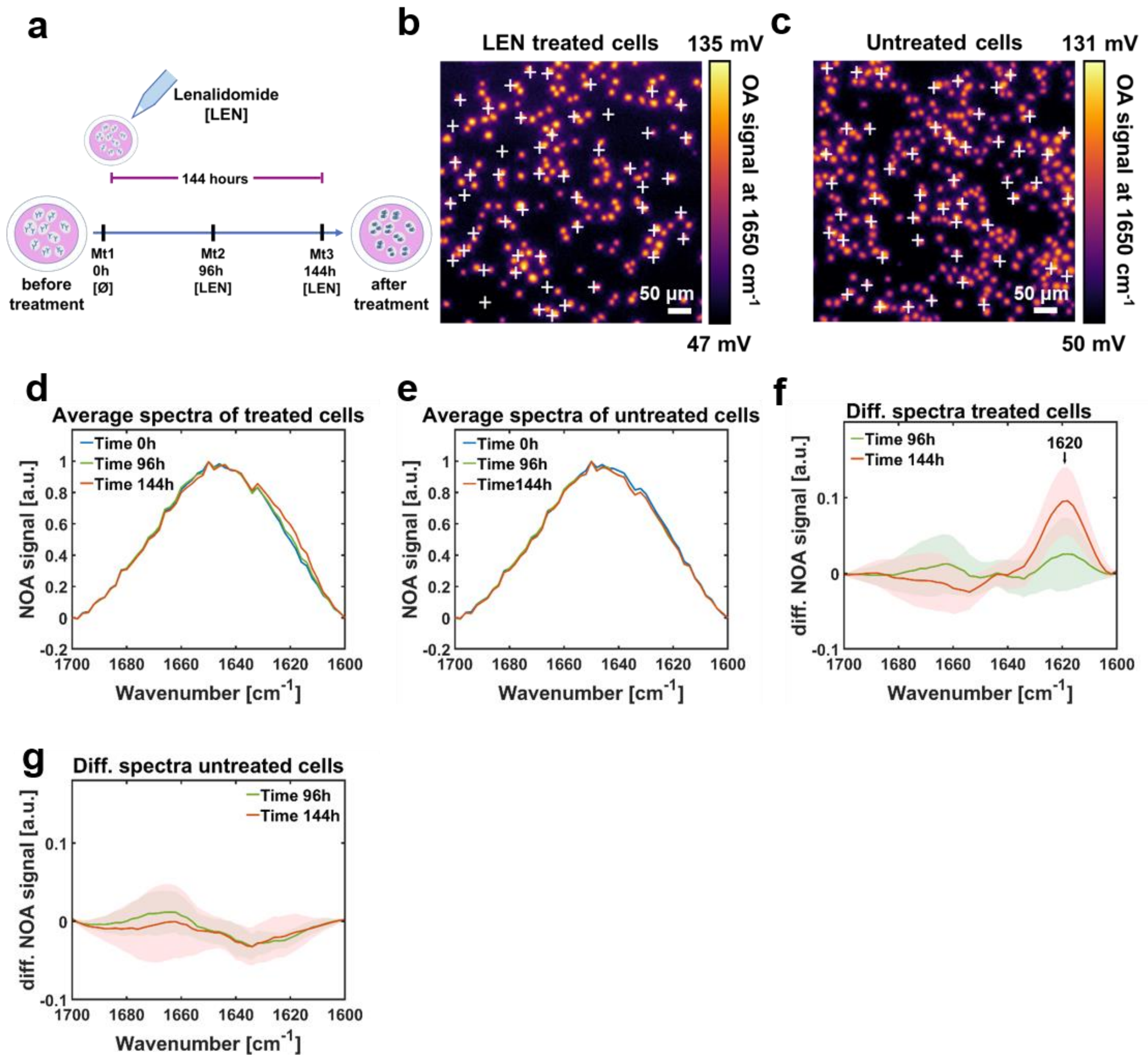

Supplementary Figure 7. Lenalidomide (LEN) treatment of MM1.S cells. a) Schematic diagram of LEN treatment of myeloma cells. **b)** MM1.S cells treated with LEN were imaged after 96 and 144 hours of treatment at 1650 cm^{-1}. **c)** For comparison, MM1.S untreated cells were imaged at 1650 cm^{-1} after 96 and 144 hours of being in culture. **d)** Comparison between the normalized average spectra of 50 myeloma cells treated with LEN at time points 0 hours, 96 hours and 144 hours. The average spectrum at 144 hours (in red) shows an enlargement towards lower frequency compared to the spectrum at 0 hours. **e)** Comparison between the normalized average spectra of 50 myeloma untreated cells at time points 0 hours, 96 hours and 144 hours. **f)** Differential spectra of LEN-treated cells at 96 hours (in green) and 144 hours (in blue). Intermolecular β-sheet structures are present in 50% of the cells after 96 hours, (green band at 1620 cm^{-1}), and in 100% of the cells after 144 hours (red band at 1620 cm^{-1}). **g)** Differential spectra of untreated cells at 96 and 144 hours. The intermolecular β-sheet band at 1620 cm^{-1} is absent in the spectra of untreated cells (n=3 independent experiments). OA – Optoacoustic. NOA – Normalized Optoacoustic.

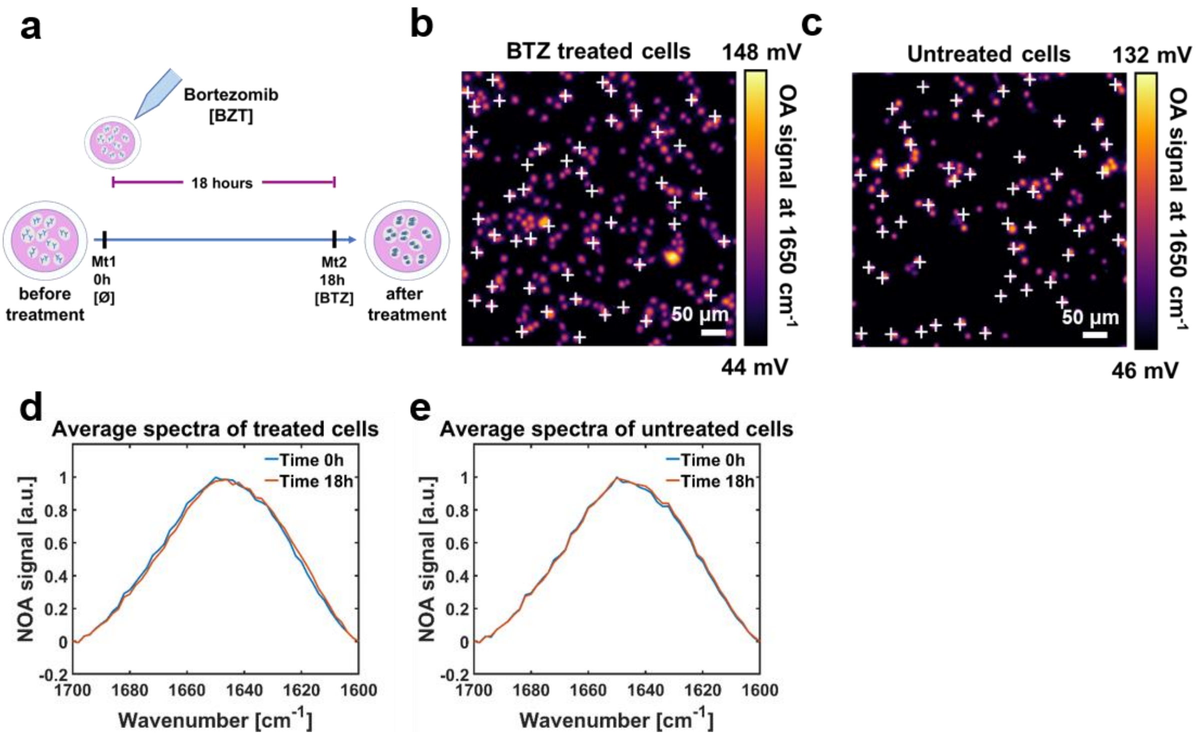

Supplementary Figure 8. Bortezomib (BTZ) treatment of MM1.S cells. a) Schematic diagram of BTZ treatment of myeloma cells. **b)** MM1.S cells treated with BTZ were imaged at 1650 cm^{-1}after 18 hours of treatment. **c)** For comparison, MM1.S untreated cells were imaged for 18 hours at 1650 cm^{-1}. **d)** Comparison between the normalized average spectra of 50 myeloma cells treated with BTZ at time point 0 hours and at 18 hours. The average spectrum acquired after 18 hours (in red) shows a small enlargement towards lower frequency compared to the spectrum acquired at 0 hours. **e)** Comparison between the normalized average spectra of 50 myeloma untreated cells at 0 and 18 hours. (n=3 independent experiments). OA – Optoacoustic. NOA – Normalized Optoacoustic.

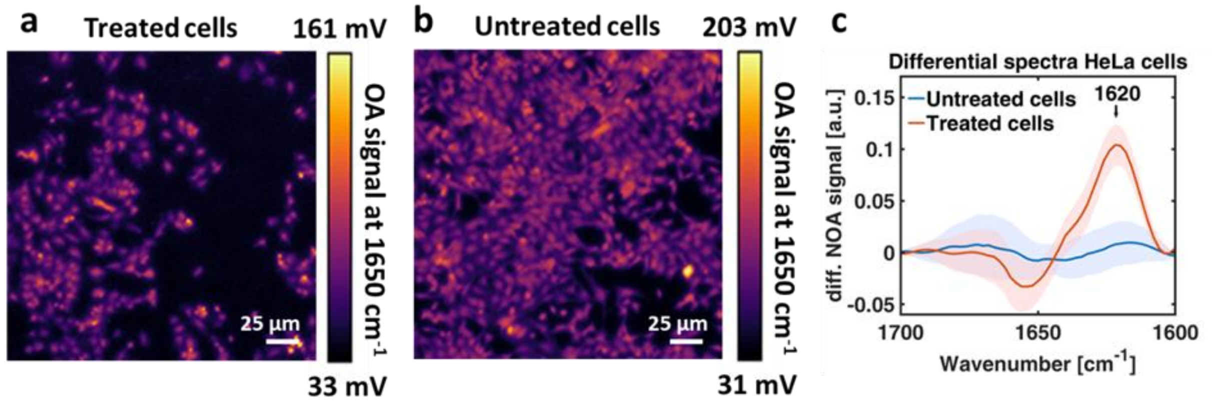

Supplementary Figure 9. HeLa cells treatment with MG132 (Carbobenzoxy-L-leucyl-L-leucyl-L-leucinal). a) Representative image of HeLa cells acquired at 1650 cm-1 after treatment with 20 µM proteasome inhibitor MG132 for 22 hours. b) Representative image of untreated HeLa cells acquired at 1650 cm-1 after 22 hours of culturing. c) Differential spectra of MG132 treated HeLa cells (red line) shown in (a), and untreated HeLa cells (blue line) in (b) at 22 hours. The spectrum in red shows a band at 1620 cm-1 assigned to intermolecular β-sheet of misfolded proteins which was not found in untreated cells. The band is similar to the band identified in the myeloma cells study (Fig. 2c).Diff.: differential NOA: normalized optoacoustic.

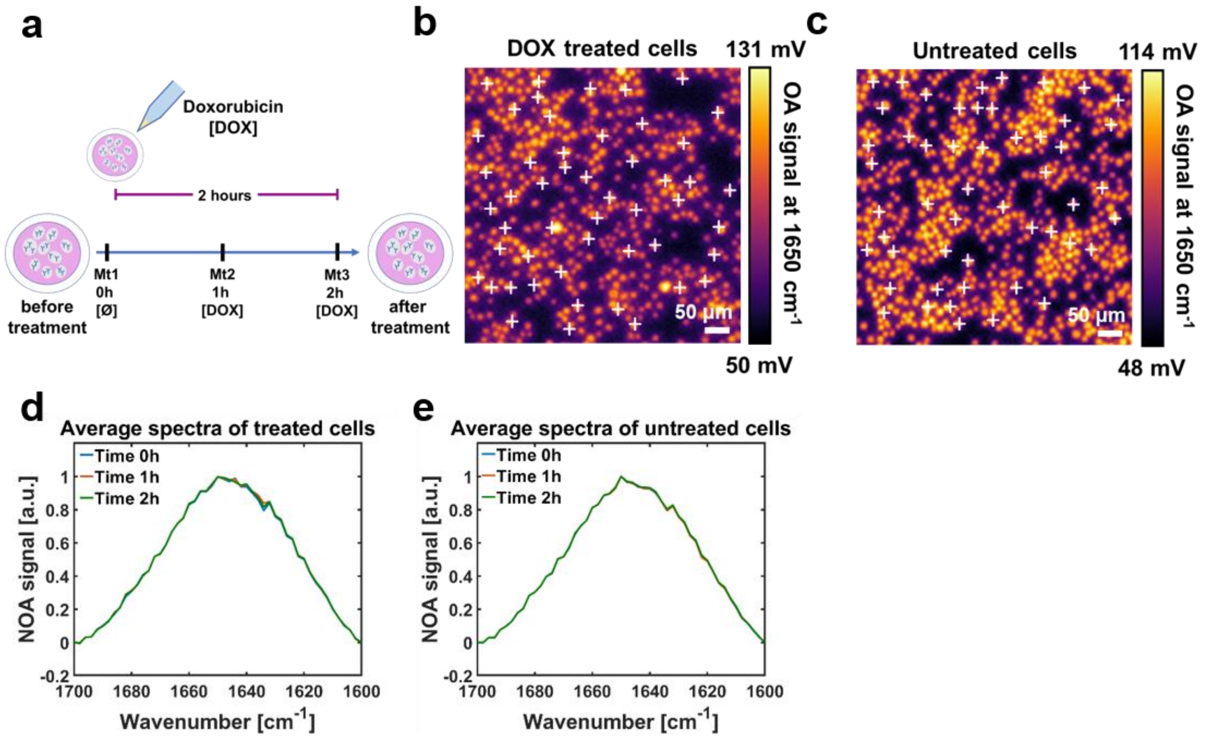

Supplementary Figure 10. Doxorubicin (DOX) treatment of MM1.S cells. a) Schematic diagram of DOX treatment of myeloma cells. **b)** MM1.S cells treated with DOX were imaged for 2 hours at 1650 cm^{-1}. **c)** For comparison, MM1.S untreated cells were imaged for 2 hours at 1650 cm^{-1}. **d)** Comparison between the normalized average spectra of 50 myeloma cells treated with doxorubicin at time points 0 hours, 1 hours and 2 hours. **e)** Comparison between the normalized average spectra of 50 myeloma untreated cells at time points 0 hours, 1 hours and 2 hours. Spectra in (d) and (e) are comparable. (n=3 independent experiments). OA – Optoacoustic. NOA – Normalized Optoacoustic.

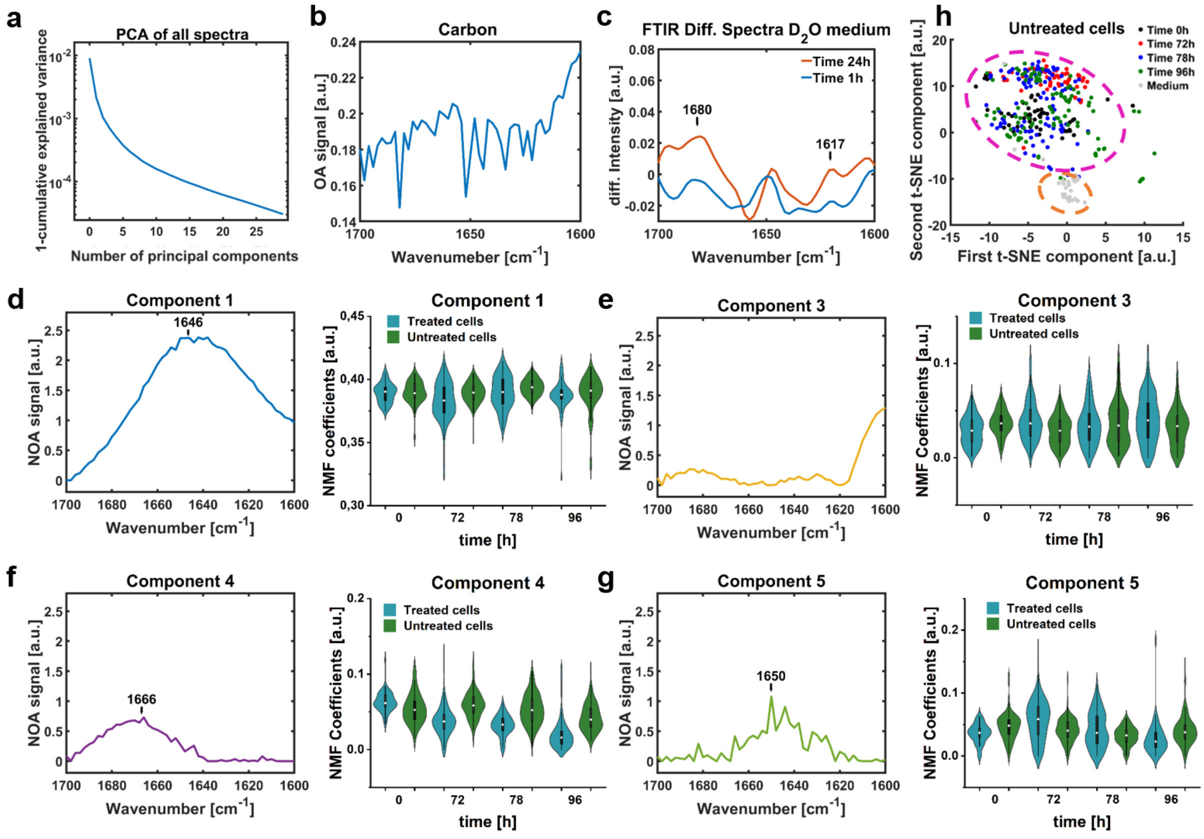

Supplementary Figure 11. Principal Component Analysis (PCA) and Non-negative Matrix Factorization (NMF) components. a) PCA of all spectra obtained from myeloma cells (MM1.S) treated with lenalidomide (LEN) and bortezomib (BTZ) and untreated MM1.S cells. **b)** Emission profile of the laser. **c)** Fourier Transform InfraRed (FTIR) spectra show a band around 1680 cm^{-1} characteristic of H/D exchange. **d-g)** NMF components and violin plots from kernel density estimate the time evolution of the components' coefficients for LEN/BTZ treated and untreated myeloma cells. **h)** A t-Stochastic Neighbor Embedding (t-SNE) map representing the distribution of all 5 components in untreated myeloma cells. (n=700 spectra). NOA – Normalized Optoacoustic.

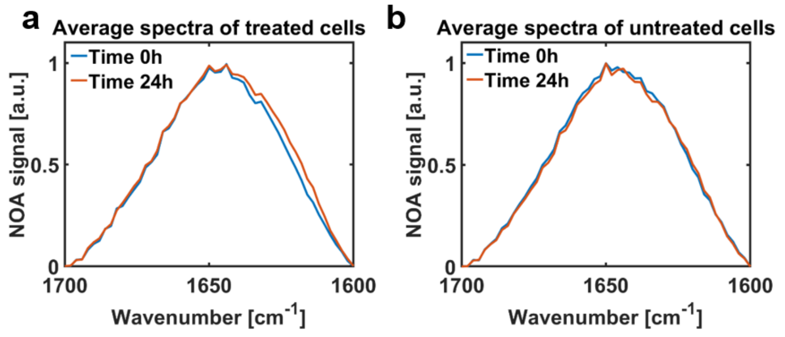

Supplementary Figure 12. Spectra of myeloma cells from patients sensitive to lenalidomide (LEN) and bortezomib (BTZ). a) Comparison between the normalized average spectra of 50 LEN and BTZ treated myeloma cells purified from a patient at time point 0 (in blue, before LEN and BTZ administration), and after 72 hours of LEN treatment and 24 h of BTZ treatment (in red). **b)** Comparison between the normalized average spectra of 50 untreated myeloma cells purified from a patient at 0 hours (in blue), and 72 hours (in red) after the medium exchange. In (a), the spectrum acquired at 72 hours shows an enlargement towards lower frequencies compared to spectrum at 0 hours, while in (b) there is no difference between the average spectra acquired at 0 hours after 72 hours in culture. (n=9 patients). NOA – Normalized Optoacoustic.

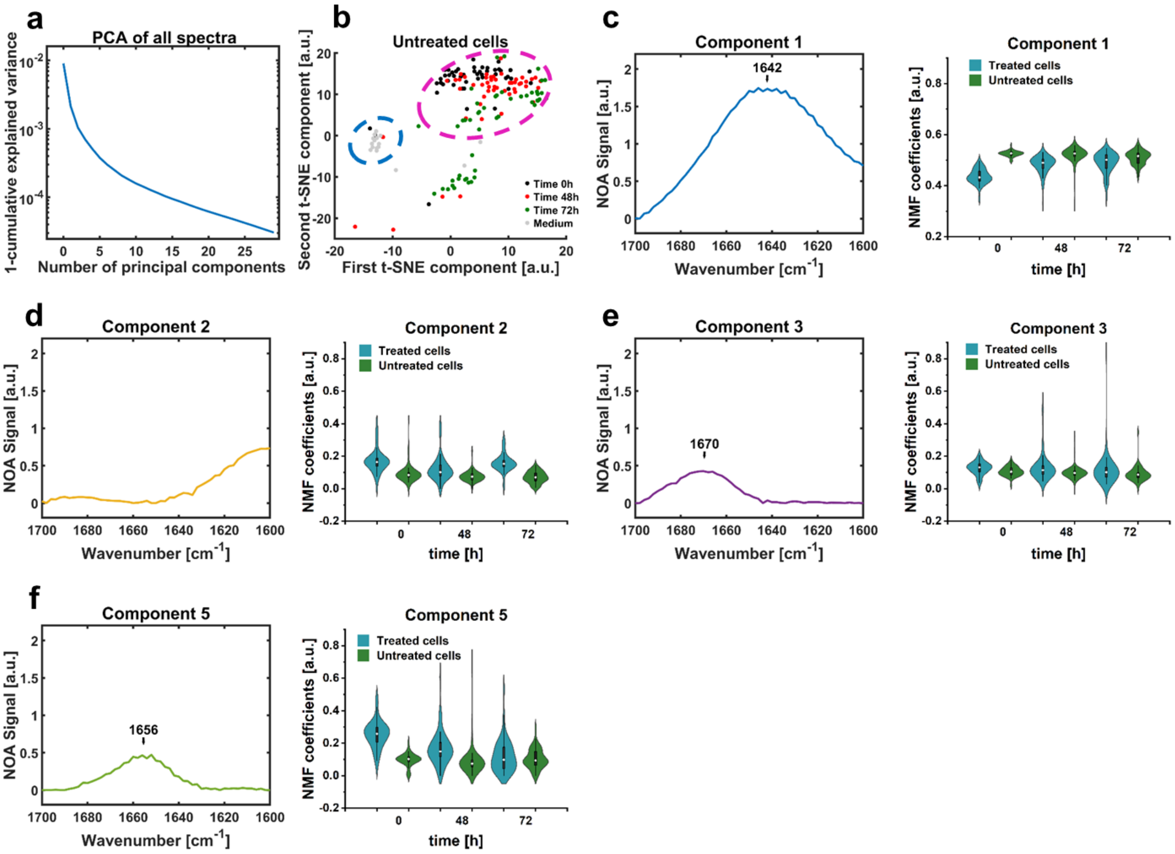

Supplementary figure 13. Image monitoring and computational analysis of untreated myeloma cells purified from a patient. a) Principal Component Analysis (PCA) performed on myeloma cells treated with lenalidomide (LEN) and bortezomib (BTZ) and untreated myeloma cells. **b)** t-Stochastic Neighbour Embedding (t-SNE) map representing the distribution of the 5 components identified by Non-Negative Matrix Factorization (NMF) in untreated cells. **c-f)** NMF components and violin plots from kernel density estimate the time evolution of the components' coefficients for LEN/BTZ treated and untreated myeloma cells. Component 1 at 1642 cm^{-1} (c) refers to the medium, component 2 (d) describes the emission profile of the laser, component 3 at 1670 cm^{-1} (e) and component 5 at 1656 cm^{-1} (f) are associated with α-helix structures. (n=400 spectra). NOA – Normalized Optoacoustic.

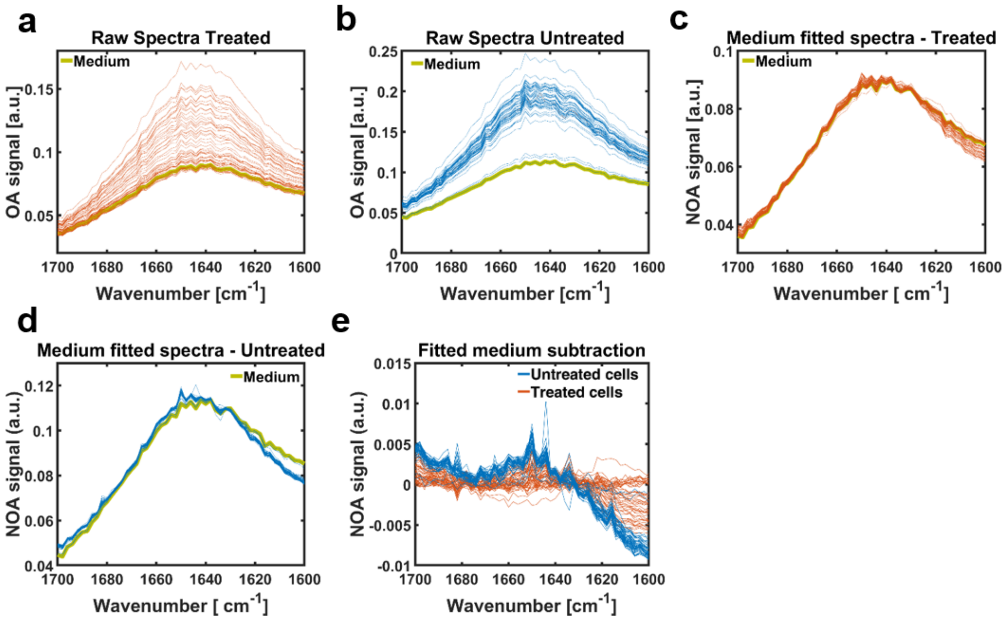

Supplementary figure 14. Least – square method. Raw spectra of lenalidomide (LEN) and bortezomib (BTZ) treated **(a)** and untreated **(b)** patient cells with the corresponding medium spectrum (in green). Spectra of LEN/BTZ treated **(c)** and untreated **(d)** patient cells fitted with their corresponding medium spectrum using the curve least squares fitting method. **e)** Spectra of LEN/BTZ treated and untreated cells after subtraction of the fitted medium spectrum. **f)** Comparison between differential spectra of LEN/BTZ treated and untreated single cells extracted from a patient sensitive to LEN and BTZ. LEN/BTZ treated cells show an increase of signal in the region of the intermolecular ß-sheet structure (1638 – 1615 cm^{-1}). OA – Optoacoustic. NOA – Normalized Optoacoustic.

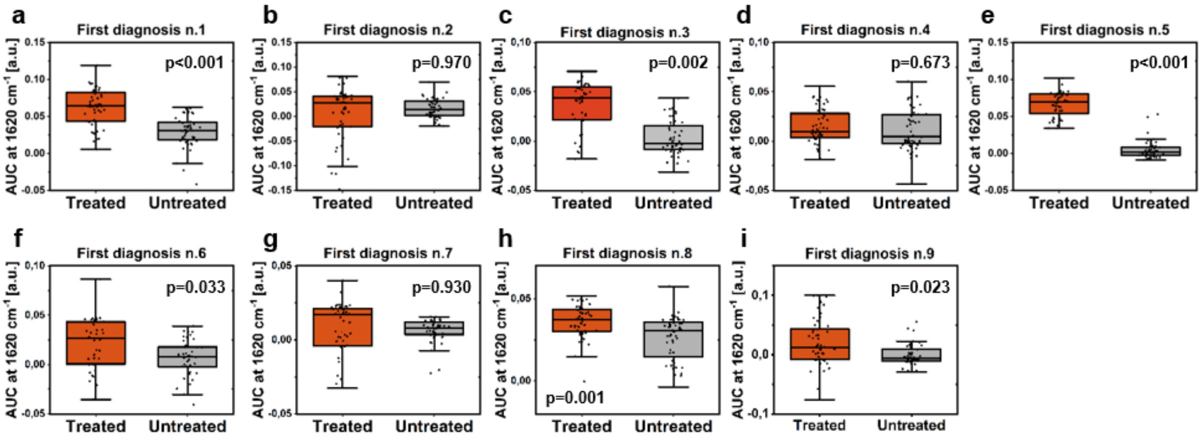

Supplementary figure 15. Single-cells response of lenalidomide (LEN) and bortezomib (BTZ) sensitive myeloma patients. a-i) Boxplot representing the Area Under the Curve (AUC) of the band at 1638 – 1615 cm^{-1} of the amide I differential spectra obtained in treated and untreated cells extracted from the bone marrow of 10 LEN and BTZ sensitive myeloma patients. The corresponding percentage responses are reported in **Table 3**. P values from a two-sided paired sample *t*-test.

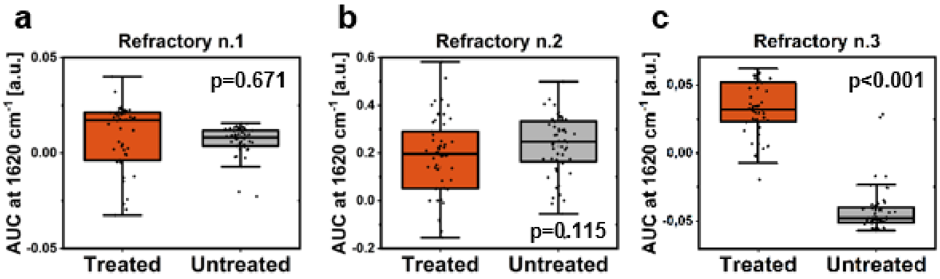

Supplementary figure 16. Single-cell response of lenalidomide (LEN) and bortezomib (BTZ) resistant myeloma patients. a-c) Boxplot representing the Area Under the Curve (AUC) of the band at 1638 – 1615 cm^{-1} of the amide I differential spectra obtained in LEN/BTZ treated and untreated cells extracted from the bone marrow of 3 patients refractory either to LEN or BTZ or both. The corresponding percentage responses are reported in **Table 3**. P values from paired sample *t*-test.

Supplementary Table 1. Relevant absorption peaks of hemoglobin, concanvalin by Attenuated Total Reflection Fourier-Transform-InfraRed (ATR-FTIR) spectroscopy and by Mid-infraRed Optoacoustic Microscopy (MiROM). The difference (Δλ) between ATR-FTIR and MiROM's values is indicated in the third column.

PROTEIN	FTIR peaks (cm^{-1})	MiROM peaks (cm^{-1})	Δλ (cm^{-1})
Hemoglobin	1649	1649	0
Concanavalin	1635	1634	1
Concanavalin	1624	1622	2
Concanavalin	1695	1691	4

Supplementary Table 2. Comparison between the peak values of different secondary structures detected in live HeLa cells by by Mid-infraRed Optoacoustic Microscopy (MiROM) and the values reported in literature.[22]

Points	β-sheet<br>(1638-1615 cm^{-1}) [22]<br>(1694-1672 cm^{-1}) [22]	α-helix<br>(1660-1642 cm^{-1}) [22]	turns<br>(1691-1653 cm^{-1}) [22]
P1	1630	1642,1651,1657	1678,1683,1688
P2	1629	1642, 1651,1656	1679,1683,1688
P3	1631	1643,1650,1658	1679,1683,1688

Supplementary Table 3. Percentage response (%) of untreated and lenalidomide (LEN) and bortezomib (BTZ) treated cells, shown per patient and calculated counting the number of single cells that show formation of β-sheet intermolecular structure during spectral imaging with Mid-infraRed Optoacoustic Microscopy (MiROM).

Patient	LEN/BTZ treated cells			Untreated cells				
First diagnosis	β-sheet band	No β-sheet	% response	β-sheet band	No β-sheet	% response	Induction regimen	Hematological response
1	47 cells	9 cells	84%	27 cells	25 cells	52%	VRD	VGPR
2	20 cells	30 cells	40%	15 cells	35 cells	30%	Dara-CyBorD	VGPR
3	32 cells	18 cells	64%	1 cells	49 cells	2%	VRD	VGPR
4	2 cells	48 cells	1%	9 cell	41 cells	18%	-	-
5	49 cells	1 cells	98%	5 cells	45 cells	10%	Dara-CyBorD	VGPR
6	15 cells	26 cells	37%	1 cells	37 cells	3%	-	-
7	22 cells	28 cells	44%	0 cells	50 cells	0%	Dara-VRD	n/a
8	37 cells	16 cells	70%	28 cells	28 cells	50%	Dara-KRD	VGPR
9	22 cells	31 cells	42%	7 cell	43 cells	14%	Dara-VRD	VGPR
**Total**			**53%**			**20%**		
Refractory	β-sheet band	No β-sheet	% response	β-sheet band	No β-sheet	% response	Re-induction regimen	Hematologial response
r/r1	0 cells	54 cells	0%	1 cell	51 cells	2%	Dara-Panobinostat-VelDex	PD
r/r2	4 cells	46 cells	8%	4 cells	46 cells	8%	VCD	PD
r/r3	18 cells	31 cells	37%	0 cells	50 cells	0%	Dara-Pom-PACE	CR

Abbreviations therapy regimens:
VRD=Bortezomib, Lenalidomide, Dexamethasone
Dara= Daratumumab
CyBorD/VCD=Cyclophosphamide, Bortezomib, Dexamethasone
KRD=Carfilzomib, Lenalidomide, Dexamethasone
Pom-PACE=Pomalidomide, Cisplatin, Doxorubicin, Cyclophosphamide, Etoposide
VelDex= Bortezomib, Dexamethasone
Abbreviations remission status:
CR=Complete remission
VGPR=Very good partial response
PD=Progressive disease
