# Supplementary material for: Label-free protein-structure-sensitive live-cell microscopy for patient-specific assessment of myeloma therapy
Source: Nat Biomed Eng. 2025 Jul 14;10(1):56–68. doi: 10.1038/s41551-025-01443-3 (PMC12823395; doi:10.1038/s41551-025-01443-3)
Supplement: Supplementary file 2 — Reporting Summary [file 41551_2025_1443_MOESM2_ESM.pdf]

nature portfolio

Corresponding author(s): Prof. Dr. Vasilis Ntziachristos, Prof. Dr. Miguel A. Pleitez

Last updated by author(s): Apr 4, 2025

Reporting Summary

Nature Portfolio wishes to improve the reproducibility of the work that we publish. This form provides structure for consistency and transparency in reporting. For further information on Nature Portfolio policies, see our Editorial Policies and the Editorial Policy Checklist.

Statistics

For all statistical analyses, confirm that the following items are present in the figure legend, table legend, main text, or Methods section.

n/a	Confirmed	
☐	☒	The exact sample size (*n*) for each experimental group/condition, given as a discrete number and unit of measurement
☐	☒	A statement on whether measurements were taken from distinct samples or whether the same sample was measured repeatedly
☐	☒	The statistical test(s) used AND whether they are one- or two-sided<br>*Only common tests should be described solely by name; describe more complex techniques in the Methods section.*
☒	☐	A description of all covariates tested
☒	☐	A description of any assumptions or corrections, such as tests of normality and adjustment for multiple comparisons
☐	☒	A full description of the statistical parameters including central tendency (e.g. means) or other basic estimates (e.g. regression coefficient) AND variation (e.g. standard deviation) or associated estimates of uncertainty (e.g. confidence intervals)
☐	☒	For null hypothesis testing, the test statistic (e.g. *F*, *t*, *r*) with confidence intervals, effect sizes, degrees of freedom and *P* value noted<br>*Give P values as exact values whenever suitable.*
☒	☐	For Bayesian analysis, information on the choice of priors and Markov chain Monte Carlo settings
☒	☐	For hierarchical and complex designs, identification of the appropriate level for tests and full reporting of outcomes
☒	☐	Estimates of effect sizes (e.g. Cohen's *d*, Pearson's *r*), indicating how they were calculated

Our web collection on statistics for biologists contains articles on many of the points above.

Software and code

Policy information about availability of computer code

Data collection	Commercially available software Matlab 2018b (Matlab, Mathworks, USA) was used for data collection.
Data analysis	Data analysis was conducted with Matlab 2019b built-in functions (Matlab, Mathworks, USA), and tools in the scikit-learn Python package (version 3.7.1). Flow cytometry data have been analyzed with FlowJo software.

For manuscripts utilizing custom algorithms or software that are central to the research but not yet described in published literature, software must be made available to editors and reviewers. We strongly encourage code deposition in a community repository (e.g. GitHub). See the Nature Portfolio guidelines for submitting code & software for further information.

Data

Policy information about availability of data

All manuscripts must include a data availability statement. This statement should provide the following information, where applicable:

- Accession codes, unique identifiers, or web links for publicly available datasets
- A description of any restrictions on data availability
- For clinical datasets or third party data, please ensure that the statement adheres to our policy

The data that support the findings of this study are available from the corresponding authors upon reasonable request.

Research involving human participants, their data, or biological material

Policy information about studies with human participants or human data. See also policy information about sex, gender (identity/presentation), and sexual orientation and race, ethnicity and racism.

Reporting on sex and gender	In total, 12 individuals with multiple myeloma were included in this study. 9 individuals were first diagnosis patients sensitive to Lenalidomide and Bortezomib treatment, while 3 individuals were relapsed-refractory patients resistant to Lenalidomide or Bortezomib, or both. We collect data from 6 females and 6 males (sex) between 35 and 72 years old. Sex and gender were not considered in the study design. In the source data disaggregated sex and gender data have not been collected. No sex and gender-based analyses were performed.
Reporting on race, ethnicity, or other socially relevant groupings	Race, ethnicity or other socially relevant grouoings were not considered in our study.
Population characteristics	The 12 individuals analysed had the following plasma cells dyscrasia: NDMM (newly diagnosed multiple myeloma, 4 individuals), NDMM+AL amyloidosis (2 individuals), MGUS (Monoclonal gammopathy of undetermined significance, 2 individuals), NDMM/POEMS syndrome (1 individual), RRMM (Relapsed/refractory MM, 3 individuals). The population examined had an age comprising between 35 and 72 years old. 6 male and 6 female individuals were involved in the study.
Recruitment	The participants were recruited at Klinikum Rechts der Isar in Munich from the specialized medical doctors. Informed consent was obtained from all individuals before bone marrow biopsy. Not self selction bias has been applied.
Ethics oversight	The study was approved by the local TUM (Technical University Munich) University Hospital ethics committee (ethical approval 672/21-S) on 17.11.2021, and written informed consent was obtained from each patient.

Note that full information on the approval of the study protocol must also be provided in the manuscript.

Field-specific reporting

Please select the one below that is the best fit for your research. If you are not sure, read the appropriate sections before making your selection.

☒ Life sciences ☐ Behavioural & social sciences ☐ Ecological, evolutionary & environmental sciences

For a reference copy of the document with all sections, see nature.com/documents/nr-reporting-summary-flat.pdf

Life sciences study design

All studies must disclose on these points even when the disclosure is negative.

Sample size	No sample-size calculation was performed. Sample size was defined by sample availability based on the ethical guidelines and regulations governing the use of human in research. The sample size (n=50 cells for time points) was chosen to restrict the measurement to 1 hours and avoid toxicity or influence of deuterated water in the measurement.
Data exclusions	No data was excluded from the analysis.
Replication	All the experiments presented here were succesfully replicate independently at least three times.
Randomization	The sample and the participants were chosen randomly. The participants were belonging to two different groups: first diagnosis patients sensitive to Lenalidomide and Bortezomib treatment and refractory or relapsed patients resistant to Lenalidomide and/or Bortezomib treatment.
Blinding	No blinding studies were performed. Blinding has not been applied due to the nature of the study requiring treatment, but the cells analyzed and the patients were randomly selected.

Reporting for specific materials, systems and methods

We require information from authors about some types of materials, experimental systems and methods used in many studies. Here, indicate whether each material, system or method listed is relevant to your study. If you are not sure if a list item applies to your research, read the appropriate section before selecting a response.

Materials & experimental systems

n/a	Involved in the study
☒	☐ Antibodies
☐	☒ Eukaryotic cell lines
☒	☐ Palaeontology and archaeology
☒	☐ Animals and other organisms
☒	☐ Clinical data
☒	☐ Dual use research of concern
☒	☐ Plants

Methods

n/a	Involved in the study
☒	☐ ChIP-seq
☐	☒ Flow cytometry
☒	☐ MRI-based neuroimaging

Eukaryotic cell lines

Policy information about cell lines and Sex and Gender in Research

Cell line source(s)	HeLa cells from American Type Culture Collection (CCL-2) and MM1.S from American Type Culture Collection (CRL-2974).
Authentication	None of the cell lines used were authenticated.
Mycoplasma contamination	All cell lines used were tested free from micoplasma.
Commonly misidentified lines (See ICLAC register)	Cell lines were used in the study

Plants

Seed stocks	n/a
Novel plant genotypes	n/a
Authentication	n/a

Flow Cytometry

Plots

Confirm that:

☒ The axis labels state the marker and fluorochrome used (e.g. CD4-FITC).

☒ The axis scales are clearly visible. Include numbers along axes only for bottom left plot of group (a 'group' is an analysis of identical markers).

☒ All plots are contour plots with outliers or pseudocolor plots.

☒ A numerical value for number of cells or percentage (with statistics) is provided.

Methodology

Sample preparation	Hela cells (ATCC: CCL-2) were cultured in DMEM medium composed by different % of D2O and MilliQ water. Cells were washed with PBS and incubated with Propidium Iodide /RNase before flow cytometry.
Instrument	Cytoflex LX (Beckman Coulter)
Software	Flowjo software (BD Bioscience)
Cell population abundance	At least 20.000 events per sample were analyzed. The percentage of Live cells was recorded as they were depicted in quadrant (Q3).
Gating strategy	Initial Gate: an initial gate (Gate P1) was created to include the cell population based on FSC vs. SSC to exclude debris.<br>Doublet Discrimination: We Used FSC-A vs. FSC-H or to gate out cell doublets and ensure single cells are analyzed (Gate P2).<br>Quadrant Gates for Annexin V/PI Staining :<br>• Annexin V-FITC vs. PI Plot:

• Q1 (Annexin V-/PI+): Necrotic cells (PI positive, Annexin V negative)
• Q2 (Annexin V+/PI+): Late apoptotic or necrotic cells (both Annexin V and PI positive)
• Q3 (Annexin V-/PI-): Live cells (both Annexin V and PI negative)
• Q4 (Annexin V+/PI-): Early apoptotic cells (Annexin V positive, PI negative)

☒ Tick this box to confirm that a figure exemplifying the gating strategy is provided in the Supplementary Information.
